# Constraining LQG Graph with Light Surfaces: Properties of BH Thermodynamics for Mini-Super-Space, Semi-Classical Polymeric BH

**DOI:** 10.3390/e22040402

**Published:** 2020-03-31

**Authors:** Daniela Pugliese, Giovanni Montani

**Affiliations:** 1 Research Centre of Theoretical Physics and Astrophysics, Institute of Physics, Silesian University in Opava, Bezručovo Náměstí 13, CZ-74601 Opava, Czech Republic; 2 ENEA- R.C. Frascati, UTFUS-MAG, Via Enrico Fermi 45, Frascati, 00044 Roma, Italy; giovanni.montani@enea.it; 3 Physics Department, Sapienza University of Rome, P.le Aldo Moro 5, 00185 Roma, Italy

**Keywords:** quantum gravity, loop quantum gravity (LQG), graphs, polymeric black hole regular black hole, Killing horizons, event horizons, stationary observers

## Abstract

This work participates in the research for potential areas of observational evidence of quantum effects on geometry in a black hole astrophysical context. We consider properties of a family of loop quantum corrected regular black hole (BHs) solutions and their horizons, focusing on the geometry symmetries. We study here a recently developed model, where the geometry is determined by a metric quantum modification outside the horizon. This is a regular static spherical solution of mini-super-space BH metric with Loop Quantum Gravity (LQG) corrections. The solutions are characterized delineating certain polymeric functions on the basis of the properties of the horizons and the emergence of a singularity in the limiting case of the Schwarzschild geometry. We discuss particular metric solutions on the base of the parameters of the polymeric model related to similar properties of structures, the metric Killing bundles (or metric bundles MBs), related to the BH horizons’ properties. A comparison with the Reissner–Norström geometry and the Kerr geometry with which analogies exist from the point of their respective MBs properties is done. The analysis provides a way to recognize these geometries and detect their main distinctive phenomenological evidence of LQG origin on the basis of the detection of stationary/static observers and the properties of light-like orbits within the analysis of the (conformal invariant) MBs related to the (local) causal structure. This approach could be applied in other quantum corrected BH solutions, constraining the characteristics of the underlining LQG-graph, as the minimal loop area, through the analysis of the null-like orbits and photons detection. The study of light surfaces associated with a diversified and wide range of BH phenomenology and grounding MBs definition provides a channel to search for possible astrophysical evidence. The main BHs thermodynamic characteristics are studied as luminosity, surface gravity, and temperature. Ultimately, the application of this method to this spherically symmetric approximate solution provides us with a way to clarify some formal aspects of MBs, in the presence of static, spherical symmetric spacetimes.

## 1. Introduction

Providing astrophysical evidence of any quantum effects on the large scales of the general relativistic (GR) geometry is a pressing and long-sought channel of analysis of the current research efforts in the development of the theory as well as in observations and data analysis. Observational feedbacks, confirming or, vice versa, diverging expectations, could provide indications, directions, or confirmations to the theory. The astrophysical phenomenology of the black hole (BH) horizons is an increasingly attractive and promising channel that has revealed itself to be surprisingly rich of applications, both on the phenomenological and theoretical level. There is great expectation concerning the two new phenomenological windows on the High Energy Universe, represented by the Event Horizon Telescope (https://eventhorizontelescope.org/) and the Gravitational Waves detection combined with data from their electromagnetic counterparts. There is hope that new data will shed light on unexpected aspects of BH theory and particularly in the near horizon geometry. This work fits into this investigation by discussing a special structure, the metric Killing bundles. We present this novel frame applied to a Loop Quantum Gravity (LQG) approximate (semiclassical) BH solution. We consider properties of the light-surfaces defined from the metrics symmetries and the associated relativistical photon orbital frequencies. The objects brought into play here, as the base of the newly introduced structures, Killing metric bundles, ground in fact many constraints of the BH (and Killing horizons) astrophysics ranging from physics of accretion to the jet emission, from BH magnetosphere to some models of Quasi-Periodic Oscillation (QPOs) emission. As a consequence of this, this frame and the new approach could reveal ample applications. The introduction of metric bundles potentially highlights small but finite discrepancies, expectably detectible from the observations of the photon orbital frequencies, and provides an alternative and new framework for the investigation of the BH geometries. One goal of this analysis is to constrain the graph bridging the quantum (discrete) and (semi-)classical (continuum) geometry with special light-surfaces. The graph, in a general LQG application, is in fact geometrical construction associated with loop quantum gravity states which can in fact be interpreted as the basis of states of a quantum (discrete) geometry, grounding from original Penrose’s spin networks. In the Penrose original combinatorial graphs, spins labelled the graph, and graphs turned to be just the mathematical (geometrical) objects used to describe the quanta of space for a spin network theory. Such original graph has been modified later in different loop inspired models. Graphs clearly have a direct application in the relational approaches, where the adjacency (links, lines) describes relations in quanta of space. Within the grounding idea of gravity geometrization, quanta of space are accordingly also gravity quanta and the texture of the spacetime structures (hence framing geometry into a relational approach where relations are embedded and translated into the graph structure). In many approaches, graphs are not dynamical but rather “generalized lattices”, polyhedron (tetrahedron) modeling 3D spaces, providing eventually an area parameter. As in this analysis, we link the area parameter of the model to the BH area through the frequencies defined with the bundles. The new frame is used to relate geometries, defined by different values of LQG derived graph parameters, by the light–surfaces frequencies. Such frequencies have replicas in different geometries of the metric family defined by different values. These replicas are present also in different points of the one geometry, connecting therefore regions close to and far away from the black hole: they connect two regions of the same spacetime, possibly revealing essential discrepancies with respect to the onset provided by the classic GR solution of reference. An observer can detect certain aspects of regions close to the BH through replicas in the distant regions. These replicas appear also related to structures emerged in different analyses and called “horizons memory” and “horizons remnants” also in naked singularity extension of the geometries.

Loop Quantum Gravity (LQG) is a non-perturbative and background-independent quantum theory of gravity. In its standard formulation, “space” is described by a spin network. The formulation of the LQG spin network is represented with a graph that is generally closed and colored (with values attached to edged or vertices of its faces according to the different realizations and LQG models). The basis states of LQG are the graphs, with (valued) graph edges and nodes (vertices), associated with irreducible SU(2) representations the first and interwiners the second. More precisely, the graph vertices represent 3-volume quanta. As the graph is fully connected, the edges are in fact the “quanta” of area $A\left(j\right)=8\pi \gamma_{BI}\sqrt{j(j+1)}$, being the half-integer *j* the *edge* value, and $\gamma_{BI}$ is the Berbero–Immirzi parameter. The model is defined by a smallest possible quanta that corresponds to the minimal (quantized) area $A_{min}$, depending on the product of the $\gamma_{BI}$ and a polymeric parameter (δ).

In this work, we focus on special loop quantum corrected (polymeric) regular black hole solutions (LBHs), a special family of spherical symmetric regular loop BH solutions, whose general relativistic limit for some values of the parameters is the Schwarzschild solution.The model developed in [1,2,3,4,5] is a metric modification outside the horizon, with the minimal area derived from LQG, and the (free) parameter δ, found from a mini-super-space LQG approximation. The metric approximation is based on quantum modifications outside the horizon assuming a regular lattice with edges of lengths $d_{b}$ and $d_{c}$, reduced then to one independent δ parameter by considering the minimal area to be the LQG minimal area. In this mini-super-space model, the LQG corrections regularize (solve) the central black hole singularity problem.

One task of this analysis is the investigation of the constraints for the graph properties by constraining the values of the main graph parameters emerging from evaluations of quantities related to the LBHs, as a minimal area parameter $a_{o}$ or eventually $\epsilon =\gamma_{BI}\delta$, or the loop mass *m* which is a function here of the polymeric parameter *P* and the ADM mass *M*. For this purpose, we use *metric Killing bundles* (or metric bundles (MBs)) which are a collection of metric solutions of the parameterized family of LBHs, characterized by certain identical properties of the light-like particles orbital frequency that can be measured by observers at infinity, informing on some properties of the geometries close to the horizons, and connecting the different metrics of the bundles. These structures define also some properties of the local causal structure and thermodynamical properties of BHs as the surface gravity, temperature, and luminosity. The LBHs geometry shares similarities with the Reissner–Nordström (RN) line tensor. We consider here this analogy in the analysis of MBs applied to LBHs.

These regular quantum corrected BHs constitute an important spacetime environment to test LQG, and to search quantum-gravity effects for the MB applications. We constrain the loop graph characteristics (minimal area, polymeric parameter) on the horizons property within characteristics locally measurable from an observer in the region $r>r_{+}$, where $r_{+}$ is the outer horizon of the LBH solution, as the orbital light-like particle frequencies, and, depending on the mass parameters, here the ADM mass *M* and the polymeric mass m(M,P), which we consider separately. The orbital frequency can be measured related to the local causal structure by an observer locally, and in a point of the *extended plane* which is a plane P−r, where the MBs are defined as curves, P is the metrics family parameter, and *r* is a radial distance. In this application, as in [6,7,8,9,10,11], the *bundles* are defined as the sets of all geometries having equal limiting light-like orbital frequency, which is also an asymptotic limiting value for time-like stationary observers (as measured at infinity). MBs are conformal invariant and can be easily read in terms of the light surfaces (LS), related to the analysis of many aspects of BHs physics, as “BH” images and several processes constraining energy extraction as the BH jet emission and jet collimation. The role of MBs is clear in the geometries with Killing horizons as the Kerr geometries, and more generally in the axially symmetric spacetimes as the Kerr–Newman (KN) geometry and Kerr–de–Sitter geometry [6,7,8,9,10,11]. The MBs definition to the spherical symmetric cases considered here for the regular BH is not immediate. Spherically symmetric BHs solutions have generally a direct astrophysical interest as limiting conditions for the spinning BHs. In [6,7,8,11], the Schwarzschild geometry in MBs analysis has been considered as limiting solution for the axially symmetric Kerr geometry or the Kerr–Newman geometry, or also the Reissner–Nordström family. Schwarzschild geometry is represented as a point on the horizon in the extended plane for all these solutions [12,13]. In this respect, the LBH metric is interesting from the MB point of view as in fact these solutions are spherically symmetric and regular BHs that are asymptotically related (in the MBs sense) to the Schwarzschild solution, allowing for clarifying bundles’ characteristics.

Below we discuss more precisely some main notions on metric bundles. Introduced in [6] to explain some properties of black holes and naked singularities (NSs), MBs establish a relation between these. Their definition was first based on the investigation of the limiting frequencies of stationary observers, and define the Killing horizons for the Kerr black holes and then extended the to other exact solutions as the cosmological Kerr–de-Sitter, the Kerr Newman spacetimes, and the limiting case of Reissner–Norström solutions [6,7,8,9,10,11]. The *metric bundles*, characterized by a particular relation between the metric parameters, are sets of geometries defined by one characteristic light-like (circular) orbital frequency $\omega_{\pm }$, which is the bundle characteristic frequency, and, in the spinning geometries, also coincides with the horizons frequencies/angular velocities. A metric bundle is represented by a curve on the so-called extended plane [6,9,10,11]. The extended plane contains the entire collection of the parameterized family of metric solutions. We can define here the *extended plane* as a plane P−r where *r* is the radial distance (in polar spherical coordinate or conveniently chosen adapted to infinity Boyer–Lindquist coordinates in the Kerr, Kerr-Newman or Kerr–de–Sitter), and P is a metric family parameter (or, eventually, a set of parameters). In the extended plane, the horizons of all BHs solutions of the family can be represented as one curve or a set of curves. In the axially symmetric spacetimes with Killing horizons, the MBs are all tangent to the horizon curve on the extended plane. Then, the horizon curve emerges as the envelope surface of the set of metric bundles. In the spherically symmetric spacetimes, MBs approach asymptotically (for some special values) the horizons. The tangency condition of MBs with the horizons’ curves characteristic of the axially symmetric Killing horizons spacetimes reduces to an approximation condition for the spherically symmetric LBH we consider here as well as the RN solutions, considered in [6] as limiting static solution of the KN. On the other hand, a special adaptation of the main idea underling the MB definitions adapted to more general horizons concepts is certainly possible.

Investigating metric bundles, we explore in an alternative way some aspects of the geometries defining the bundle as measured by an observer at infinity. The metric bundle concept can be significant in the study of BH physics, in the interpretation of NSs solutions and BH thermodynamics. In this work, we present the definition of metric bundles and discuss their properties also in the context of loop BH thermodynamics.

These structures essentially explicate some properties of the Killing horizons in the axially symmetric spacetimes and events horizons in the spherically symmetric case. The horizons in this last case are limiting surfaces of the MBs in the extended plane. Each geometry of the set has, at a certain radius *r*, equal characteristic bundle frequency.

MBs are characterized by several special properties: the horizons remnants, structures of the bundles typical of certain NSs, the horizons replicas, and the idea of an “horizon memory” firstly introduced in [6] provide through the MBs a different perspective to explore these spacetimes, conferring a global vision including the possibility to study the transition of one geometry evolving in a different solution of the family. In this sense, the extended plane, where bundles are defined as curves, is endowed with a “certain plasticity” (whose typical expression is for example in the NSs remnants). Each spacetime in one point has some properties that are replicated in different geometries (BH or NSs), which can then be thought of as target or transition state in the geometry evolution as regulated by the (first law of classic) BH thermodynamics. Killing bottlenecks appear in certain NSs as restriction of the Killing throat in the associated light surfaces’ analysis. These structures (in general close to the extreme Kerr or KN solutions) were seen also as “horizons remnants” in NSs [6,7,8,9,10,11] and appear also connected with the concept of pre-horizon regime introduced in [14,15,16,17,18,19,20]. The pre-horizon was analyzed in [14,15,16,17,18,19,20]. In these analyses, it was concluded that a gyroscope would conserve a memory of the static or stationary initial state, leading to the gravitational collapse of a mass distribution [14,15,16,17,18,19,20,21,22,23,24].

The article plan: This article is structured as follows: In Section 2, we discuss the main properties of the LQG metric and metric bundles: the black hole solutions are introduced in Section 2.1, metric bundles are discussed in Section 2.1.1 and we construct the extended plane for these solutions in Section 2.1.2. The comparison with the case of the Reissner–Norström geometry is addressed in Section 2.2. Metric bundles of the LBHs are the focus of Section 3. In Section 4. We review some aspects of the BH thermodynamics exploring in Section 4.1 the BHs surfaces’ gravity, the luminosity and the temperature in terms of the loop model parameters, then these quantities are considered on metric bundles. In Section 5, we summarize the main steps of this analysis, concluding this article.

Throughout this work, we introduced a number of symbols and notations necessary to explain all the results obtained for these recently introduced objects; however, there is in fact a relatively small set of objects constituting a core of the MBs we analyze along this analysis, and are listed for reference in Table 1.

## 2. On LBHs and the Metric Bundles

In this section, we discuss the main properties of the LQG metric and the metric bundles. We introduce the LBHs solutions in Section 2.1. In Section 2.1.1, we discuss some general properties of metric bundles. Constructing the extended plane for these solutions, we compare with the case of the Reissner–Norström geometry in Section 2.2. More information on the issues related to BHs solutions in LQG can, for example, be found in [25,26,27,28], while definition of metric-bundles in the context of geometries with Killing horizons that is the other aspect underlying this analysis we refer to [6,7,8].

### 2.1. The Metric

We consider the static spherical loop BH (LBH) solution derived as LQG approximation as

(1) $$\begin{array}{c} ds^{2}=-G\left(r\right)dt^{2}+\frac{dr^{2}}{F(r)}+H\left(r\right)d\Omega^{2},\;where\;d\Omega^{2}\equiv d\theta^{2}+\sigma d\phi^{2},\;\sigma \equiv \sin^{2}\theta ,\\ G\left(r\right)\equiv \frac{\left(r-r_{+}\right)\left(r-r_{-}\right){\left(r+r_{*}\right)}^{2}}{r^{4}+a_{o}^{2}},\;H\left(r\right)\equiv r^{2}+\frac{a_{o}^{2}}{r^{2}},\;F\left(r\right)\equiv \frac{\left(r-r_{+}\right)\left(r-r_{-}\right)r^{4}}{{\left(r+r_{*}\right)}^{2}\left(r^{4}+a_{o}^{2}\right)}\;\mathrm{and} \end{array}$$

(2) $$\begin{array}{cc} & r_{*}\equiv \sqrt{r_{+}r_{-}}=2mP,\;P\left(\epsilon \right)\equiv \frac{\sqrt{1+\epsilon^{2}}-1}{\sqrt{1+\epsilon^{2}}+1},\;M=m{\left(1+P\right)}^{2}\;a_{o}=\frac{A_{min}}{8\pi }. \end{array}$$

In the metric, *P* is the polymeric parameter, where generally P≪1 so $\left(r_{-},r_{*}\right)$ vanishes and the metric can be considered approaching a Schwarzschild limit. $a_{o}$ is the area parameter, equal to $A_{min}/8\pi$, $A_{min}$ being the minimum area appearing in LQG (minimum area gap of LQG), which can be related to parameter $\epsilon =\gamma_{BI}\delta$, and $\gamma_{BI}$ is the Barbero–Immirzi parameter. *M* is the ADM mass in the Schwarzschild limit, i.e., the mass for an observer at asymptotically flat infinity; vice versa, the *m* parameter depends on the polymeric function *P* and *M*.

There are two horizons $r_{+}$ and $r_{-}$ respectively:

(3) $$\begin{array}{c} \mathrm{Horizons}:\;r_{+}=2m,\;r_{-}=2mP^{2}. \end{array}$$

**Figure 1:**
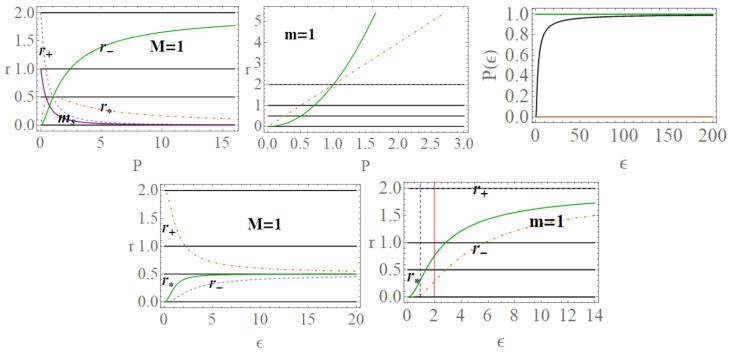
Upper left panel: horizons $r_{\pm }$ and radius $r_{*}$ of Equation (3) function of the *P* polymeric metric parameter in the terms of the LQG mass parameter *m* considered as a function of *P* (thus the notation $m_{s}$), it is here M=1 (*M* is the ADM mass in the Schwarzschild limit). Upper center panel: $r_{\pm }$ and $r_{*}$ function of the *P* for m=1, colors notation follow correspondent left panel. Upper right panel: *P* as a function of ϵ (a metric polymeric parameter) as in Equation (2). Bottom panels: horizons and radius $r_{*}$ for M=1 and m=1 respectively as functions of ϵ. Notes on notation can be found in Table 1.

**Figure 2:**
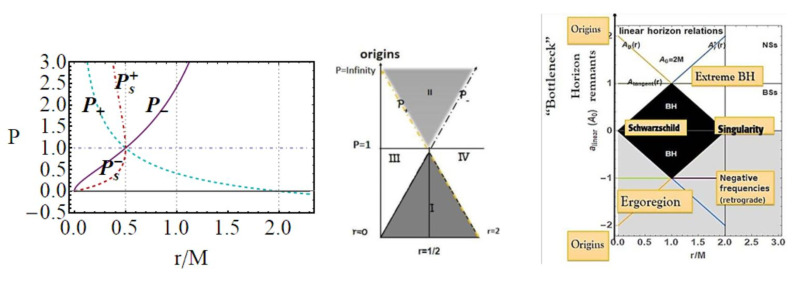
Extended planes in the Kerr geometries and LQG BHs. Left panel: $P_{\pm }$ are the horizon curves in the extended plane considered in Equation (4). Center panel: extended plane of the LBH geometry in the *P*-parametrization (*P* is the polymeric parameter). Details are in Section 2.1.2. Right panel: extended plane of the Kerr geometry, details are in [6] and Section 2.1.2; here, we point out the analogies with the extended plane structures in the two planes—see also Figure 3 and Figure 4 and Table 1.

In the following, we consider the two Killing fields of the geometry $\left(\xi_{t},\xi_{\phi }\right)$. Here, we adopt two approaches considering (**1**) m=1 (loop BH mass equal one) or (**2**) ADM mass parametrization: M=1. Clearly, M=m and $r_{+}=2m=2M$ in the (Schwarzschild) limit of P≈0. When considering the (**2**)-asset, we shall use explicitly m(M,P) to signify the dependence on *P* and *M*. Condition (1) implies $P=+\sqrt{M}-1$, condition (**2**) implies $m=1/{\left(1+P\right)}^{2}$, see Figure 1. More generally, we consider $P=\{m,P,\epsilon ,a_{o}\}$ as the geometry parameters, considering differently related (The issue of the independence of the parameters $P=\{P,m,a_{o}\}$ is deep and concerns the specific approximated BHs model as well as the LQG. Being our analysis adapted to the context of the applications of the MBs, we have adopted a more general approach, using Equation (2) in fact as particular cases. The use of m=1 implies a re-parametrization in terms of *M* which here sets the scales in some analysis. However, within the condition m=1, there is $P\to \sqrt{M}-1$, and $r_{+}=2$ (in mass units) see Figure 1—see, for example, [1]. We consider different approaches and conditions to evaluate the MBs for this geometry.). For very large ϵ, considering function P(ϵ) of Equation (2), there is $r_{\pm }=r_{*}=M/2$ (and 2m) for very small ϵ, $r_{+}=2M\left(2m\right)$ while $r_{-}=r_{*}=0$—see Figure 1 and Figure 2.

Note that here *r* is only asymptotically the usual radial coordinate since H(r) is not $r^{2}$. Figure 4 show the curves $\sqrt{H(r)}=$ constant in the plane $\left(r/M,a_{o}\right)$ and the extreme curve for the $\sqrt{H(r)}$ as a function of *r*. The function of *r* has an extreme $r_{a_{o}}^{min}$, a minimum, notably equal to the length from the minimal loop area i.e., $r_{a_{o}}^{min}=\sqrt{a_{o}}$, where $\sqrt{H(r_{a_{o}}^{min})}=2r_{a_{o}}^{min}$. Concerning the structure of the geometry and singularity nature and relative discussion of the Penrose diagram, we refer to [1,29]: geometry at r=0 has no singularity, but, in the limiting Schwarzschild case, and in fact this is a regular BH solution. The analysis of Penrose diagram shows that there is another asymptotically flat Schwarzschild region, i.e., there are two horizons and two pairs of asymptotically flat regions.

#### 2.1.1. On the MBs, Horizons, and Observers

The analysis of the metric bundles and the geometry properties with MBs focus on the properties an observer could measure in the region outside the (outer) horizon $r_{+}$ in the BH spacetime. The observer could extract information (locally) of the region close to the (inner and outer) horizons $r_{\pm }$—and connecting different geometries of the metric family considering local properties of causal structure with the analysis of photon-like orbits. In this sense, the horizon’s confinement and the ***horizon’s replicas***.

- **Definitions of horizon’s replicas and confinement**
  Considering a generic property ${℘}_{\pm }$ of the horizon as distinguished in the extended plane, as the horizon frequency ω for the spinning BH horizons, there is a *replica* of the horizon, in the same spacetime when there is an orbit (radius) $r_{x}>r_{•}$ such that $℘\left(r_{•}\right)\equiv {℘}_{•}=℘\left(r_{x}\right)$, where $r_{•}$ is a point of the horizon curve in the extended plane. From MB definition, there are horizons replicas in different geometries, i.e., there are a $p\neq p_{x}$ and a $r_{x}>r_{+}$, where *p* and $p_{x}$ are values of the extended plane parameter P, corresponding to two different geometries (distinguished with two horizontal lines of the extended plane) such that: $℘\left(r_{•}\left(p\right),p\right)\equiv {℘}_{•}^{p}=℘\left(r_{x}\left(p_{x}\right),p_{x}\right)$. In both points, $\left(r_{•},r_{x}\right)$, there is equal light-like orbital frequency. Vice versa, the (MBs’) ***horizon confinement*** is interpreted as the presence of a “*local causal ball*” in the extended plane, which is a region of the extended plane P−r, where MBs are entirely confined, this means that there are no horizons replicas in any other region of the extended plane, in any other geometry, although we can be interested in specifying this definition to confinement of the *℘* property in the same geometry. Typically, for the Kerr spacetime, the causal ball is a region upper bounded in the extended plane by the a portion of the horizon curve corresponding to the a set of the inner horizon BHs—[6,9,10,11]. The analysis of self-intersections of the bundles curves on the extended plane, in the same geometry (horizon confinement) or intersection of bundles curves in different geometries is therefore an important point of the MBs analysis. (It is obvious that, in the spherically symmetric spacetime, the definition of replica is adapted to the frame of the MBs approximation to the horizon curve in the extended pane, i.e., $r_{•}\approx r_{\pm }$). We precise the definition of the MBs by considering explicitly the definition for the Kerr spacetimes; in this discussion, it is easier to consider explicitly the definition for the metric bundles adapted to the more general axially symmetric case as in [6,7,8,9,10,11]). Therefore, the Kerr horizons are null surfaces, $S_{0}$, whose null generators coincide with the orbits of a one-parameter group of isometries; thus, there exists a Killing field L that is normal to $S_{0}$. MBs satisfy the condition $L_{N}\equiv L\cdot L=0$, where L is a Killing field of the geometry $L\equiv \partial_{t}+\omega \partial_{\phi }$. In BH spacetimes, this Killing vector defines also the thermodynamic variables and the Killing horizons. Therefore, *metric bundles* are solutions of the zero-norm condition $L_{N}\left(\omega_{•}\right)=0$ ($\omega_{+}=\omega_{•}$ for the outer horizon $r_{+}$). The condition $L_{N}=0$ is related to the definition of stationary observers, characterized by a four-velocity of the form $u^{\alpha }\propto L^{\alpha }$. The spacetime causal structure of the Kerr geometry can be then studied by considering also stationary observers [30]: timelike stationary observers have orbital frequencies (from now on simply called frequencies) in the interval $\omega \in ]\omega_{-},\omega_{+}[$ having limiting orbital frequencies, which are the photon orbital frequencies $\omega_{\pm }$, which, evaluated on the Kerr horizons $r_{\pm }$, provide the frequencies $\omega_{\pm }$ of the Killing horizons. In general, a Killing horizon is a light-like hypersurface (generated by the flow of a Killing vector), where the norm of a Killing vector is null. The event horizons of a spinning BH are therefore Killing horizons with respect to the Killing field $L_{H}\equiv \partial_{t}+\omega_{H}\partial_{\phi }$, where $\omega_{H}$ is in general angular velocity of the horizons. (The event horizon of a stationary asymptotically flat solution with matter satisfying suitable hyperbolic equations is a Killing horizon). Conditions on $\omega_{H}=$ constant represent the BH rigid rotation. For static (and spherically symmetric) BH spacetimes, the event, apparent, and Killing horizons with respect to the Killing field $\xi_{t}$ coincide. In the limiting case of the static Schwarzschild spacetime or the Reissner Nordström spacetime, the event horizons are Killing horizons with respect to the Killing vector $\partial_{t}$.
- **MBs and thermodynamics:** In this work, we also investigate some BHs thermodynamics properties of the LBHs in the extended plane through the analysis of MBs. The BH Killing horizons of stationary solutions have constant surface gravity (zeroth BH law-area theorem): the norm $L_{N}$ of L is constant on the BH horizon. Moreover, the BH surface gravity, which is a conformal invariant of the metric, may be defined as the rate at which the norm $L_{N}$ of the Killing vector L vanishes from outside ($r>r_{+}$). For a Kerr spacetime, the surface gravity re-scales with the conformal Killing vector, i.e., it is not the same on all generators, but, because of the symmetries, it is constant along one specific generator. More precisely: the constant $\kappa :\nabla^{\alpha }L=-2\kappa L^{\alpha }$, evaluated on the *outer* horizon $r_{+}$, defines the BH surface gravity, i.e., $\kappa_{+}\equiv \kappa \left(r_{+}\right)=$ constant on the orbits of L (equivalently, we can write $L^{\beta }\nabla_{\alpha }L_{\beta }=-\kappa_{+}L_{\alpha }$ and $L_{L}\kappa_{+}=0$, where $L_{L}$ is the Lie derivative—therefore defining a non-affine geodesic equation). The BH surface area is non-decreasing (second BH law); consequently, the impossibility to achieve by a physical process a BH state with zero surface gravity. More precisely, non-extremal BH cannot reach an extremal case in a finite number of steps—third BH law: at the extreme case for the Kerr geometry a=M, the maximum of the horizon curve in the extended plane, where $r_{\pm }=M$, the surface gravity is zero and, consequently, the temperature is $T_{\mathrm{BH}}=0$, but not its entropy (and therefore the BH area).(This fact poses constraints also with respect to the stability against Hawking radiation) The mass variation, the surface gravity, and the horizons frequencies are related by the first law of BH thermodynamics, which can be written as $\delta M=\left(1/8\pi \right)\kappa_{+}\delta A_{\mathrm{BH}}+\omega_{H}\delta J$, where there is the variation of the BH mass, the horizon area and angular momentum *J*, for the Kerr (BH), representing the “work term”, $A_{\mathrm{BH}}$ is the BH area.

#### 2.1.2. The Extended Plane

**Figure 3:**
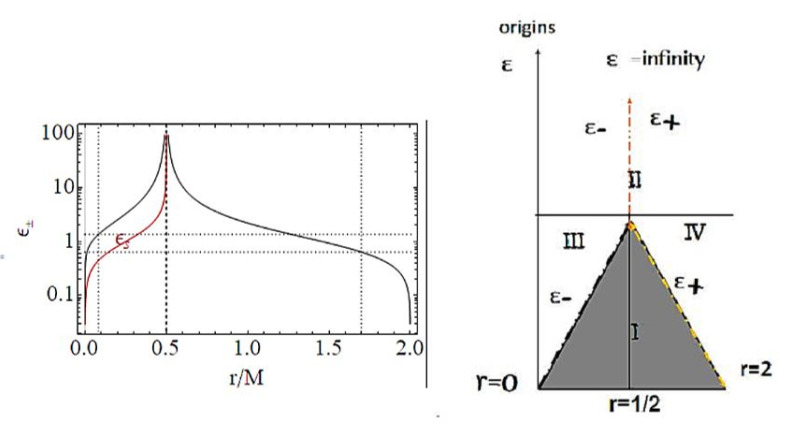
Extended plane in the ϵ−r parametrization. The horizon’s curve $\epsilon_{\pm }$ of Equation (6) in the extended plane ϵ−r is also represented together with the asymptote r=0.5M. Saddle points are horizontal dotted lines. $P_{s}^{\pm }$ curves of Equation (5) are also shown (*M* is the ADM Schwarzschild mass, ϵ, and *P* are polymeric metric parameters). Details are in Section 2.1.2—see also Figure 2 and Table 1.

In Figure 2 and Figure 3, two realizations of the extended plane of the regular LBH geometry of Equation (1) are represented. The first step to construct the extended plane P−r where the MBs are defined, is to individuate the more convenient parameter P; the second step in this construction is to represent the horizon curve in the plane. (It is clear that here a plane is briefly intended as a flat, two–dimensional surface, as in Figure 2). The main convenient parametrization for the extended plane as emerging from the analysis of MBs in Section 3 is *P*-parametrization or equivalently ϵ-parametrization, obtained by using Equation (2) and here showed in Figure 2. (More generally, here we note that, as discussed in [6,9,10,11], the first step to explore the metric bundles is to distinguish a leading parameter for the metric bundles, which in the Kerr spacetime was the dimensionless spin a/M of the singularity (a BH or NS), as clearly connected to the characteristic frequency. This is obviously determined by the Killing horizons’ definitions. This choice is, however, not immediate. In the case of Kerr-Newman (KN) spacetimes for example, as discussed in [6], one could choose spin a/M or the dimensionless electric change Q/M or, for example, the “total charge” $Q_{T}\equiv \sqrt{{\left(Q/M\right)}^{2}+{\left(a/M\right)}^{2}}$).

**Figure 4:**
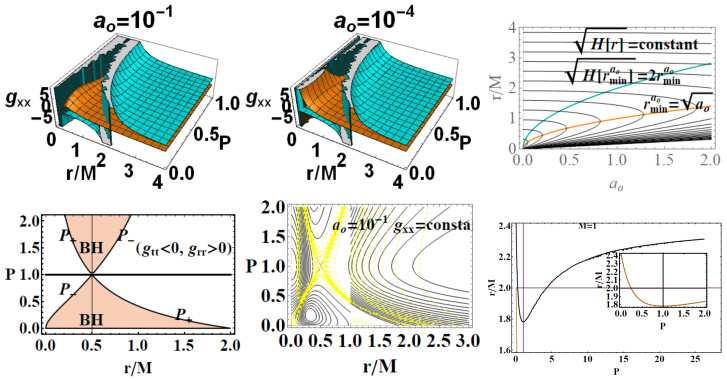
Analysis of metric (1) and metric bundles. 3D Plots show $g_{xx}$ for x∈{t,r} as a function of r/M (ADM Schwarzschild mass M=1) and polymeric metric parameter *P*, for different values of $a_{o}$ (length from the minimal LQG area). Orange surface is $g_{tt}$. The Upper Right panel shows the metric component $\sqrt{H(r)}=$ constant in the plane $\left(r/M,a_{o}\right)$, $H\left(r\right)=g_{\phi \phi }/\sigma$ in Equation (3), $\sigma \equiv \sin^{2}\theta$. Curve $r_{a_{o}}^{min}$ is the extreme curve for the $\sqrt{H(r)}$ as a function of *r*. Note that the extreme, a minimum, is equal to the length from the minimal area $r_{a_{o}}^{min}=\sqrt{a_{o}}$, and the function $\sqrt{H(r_{a_{o}}^{min})}=2r_{a_{o}}^{min}$. Bottom Left panel: extended plane P−r. Region $g_{tt}>0$ and $g_{rr}<0$ (pink-BH region, we do not consider the region P>1) and outer region is $g_{tt}<0$ and $g_{rr}>0$. Horizons $P_{\pm }$ is shown. Regions do not depend on a $a_{o}$ parameter. Line r=1/2 is also shown; this is an M=1 approach. Bottom center panel: curves $g_{xx}=$ constant for x∈{t,r}. Bottom right panel: The curve r(P) such that $g_{tt}=c_{t}=$ constant and $g_{rr}=c_{r}=$ constant; in other words, the families (in terms of *P* parameters) of metric solutions having equal $g_{tt}$ and $g_{rr}$ in the *same* point *r*. The inside plot is a zoom in the region P∈[0,1]. See also Table 1 for further details on notation.

A second issue in the plane construction is the choice of the axis *r*, which is here the radial coordinate *r*, especially relevant in the case of LBH of Equation (1). As we discussed in Section 2.1, concerning the interpretation of the radial coordinate *r* for metric Equation (1), the radius $\tilde{r}\equiv \sqrt{H[r]}$ only asymptotically approaches the standard radial coordinate, i.e., *r* is only asymptotically the usual radial coordinate (For this reason, one could think of selecting $\tilde{r}$ instead of the asymptotic standard Schwarzschild radial coordinate *r* which is the circumferential radial coordinate of the Schwarzschild geometry (from the integration around a full circle at radius *r*, we get a circumference of 2πr, i.e., the surfaces at fixed *t* and *r* appear in GR as round spheres where $ds^{2}=d\Omega^{2}$ is the standard Riemannian metric on the (unit radius) two sphere or $d\Omega^{2}$ is an interval of spherical solid angle in standard spherical coordinates (θ,ϕ)—for a fixed *r*, the surface area the circle $4\pi r^{2}$, and the associated sphere with Gaussian curvature $1/r^{2}$). The line element on an (equatorial) circle is, in the GR geometry, $ds^{2}=r^{2}d\phi^{2}$, vice versa in our case, metric (1), we shall have $ds^{2}={\tilde{r}}^{2}d\phi^{2}$. In this regard, we also note here that, as in general relativity, we could adopt the Eddington–Finkelstein coordinates adapted to radial null geodesics. In [11], however, we explicitly use MBs, based on null circular orbits, to rewrite the line element.) with respect to the reference GR solution, as H(r) is not just $r^{2}$. In this respect, in Figure 4, we represent the $\sqrt{H(r)}$ asymptotical behavior for large distance from r=0 (the line defining the bundles origins) and particularly the value $\sqrt{H(r)}=r$. We have represented both the asymptotic behavior and the curves $\sqrt{H(r)}=$ constant (versus r= constant) in the plane $\left(r/M,a_{o}\right)$ and the extreme points curve for the $\sqrt{H(r)}$ as a function of *r*. In fact, $\sqrt{H(r)}\equiv \tilde{r}$ remarkably is not monotone in *r* but has a minimum, $r_{a_{o}}^{min}=\sqrt{a_{o}}$, where $\sqrt{H(r_{a_{o}}^{min})}=2r_{a_{o}}^{min}$. Furthermore, in Figure 4, we can see the situation for different geometries (line r= constant) and at fixed geometry ($a_{o}=$ constant), where there are two orbits *r* with equal values of $\tilde{r}$. The main point of the construction of the extended plane consists in the fact that it allows for considering the properties of a parameterized family of geometries in a “global” prospective, by considering different features as seen at variation of the metric family parameter P. This turns out, therefore, to also be relevant for the exploration of the transformations leading from one solution to another of the family, as, for example, after a (dimensionless) spin shift of Kerr BH, a shift of the dimensionless electric charge in the RN metric, or eventually a variation of loop parameters P for the LBHs. Thus, in this “global” frame, we also search for a replica of the horizons of one geometry in different solutions, investigating the presence of horizons’ characteristics of different spacetimes of the family (or MBs). In this way, we also relate different geometries through their local causal properties and also BHs’ thermodynamical characteristics. These aspects are here explored in dependence on the model parameters P and evaluated on the metric bundles in Section 4.

Metric (1) shares several similarities with the Reissner–Nordström geometry, considered here in Section 2.2. To discuss some properties of the extended plane and the MBs, it is convenient, however, to refer first to the representation of the extended plane of the axially symmetric, stationary, vacuum Kerr solution. The Kerr geometry is a well-known, exact, asymptotically flat solution of Einstein equations—[6,7,8,11,12,13]. In Figure 2, the panel on the Kerr extended plane shows the negative region corresponding to counter-rotating orbits i.e., to photon orbital frequency equal to the horizon frequencies in magnitude. In the spherically symmetric cases, we can restrict our analysis, without loss of generality, to the upper part of the plane, related to the positive frequencies. Line $A\equiv a\sqrt{\sigma }=0$, corresponding to line P=0 of the plane P−r, is the Schwarzschild limiting case, where *a* is the dimensionless spin, $\sigma \equiv \sin^{2}\theta$ in Boyer–Lindquist coordinates. The origin (*line of bundles origins*) is here $A_{0}\equiv a_{0}\sqrt{\sigma }$, corresponding to P- line (r=0) of the P−r plane. The Kerr geometry ergoregion (in the extended plane) is the strip r<2M (the ergoregion of the Kerr geometry is bounded by and outer ergo-surface $r_{\epsilon }^{+}\geq r_{+}$, where $r_{+}\left(a\right)<2M$ is the outer horizon and the inner ergo-surface $r_{\epsilon }^{-}\left(0\right)\leq r_{-}$, $r_{-}$ is the inner horizon, where only on the equatorial plane is there $r_{\epsilon }^{+}=2M$ independently from the spin *a*). Extreme Kerr BH is for A=1, in panel regions for Kerr BHs or Kerr naked singularities (NSs) are also reported. For the Kerr MBs, it is relevant to consider the polar angle θ, in $\sigma \equiv \sin^{2}\theta$; in the spherical symmetric case considered here, we can consider σ=1. The extended plane of the Kerr geometry is constructed considering different functions (including the tangent curves to the horizon), ${\left\{A_{x}\right\}}_{x}$, which are given in [6,11] (they correspond also to the linearized horizons relations $r_{+}\left(r_{-}\right)$ in an equivalent extended plane). The extended plane of Kerr geometries and LBHs in Figure 2 show clear analogies. We consider an extended plane realization in Figure 1 for the LBHs. We can consider the vertical and horizontal lines and the horizons curves $P_{\pm }$ in Figure 2 as a function of *r* (for M=1). In the P−r plane (M=1), the horizons are determined by the polymeric function as

(4) $$\begin{array}{cc} & P_{+}\equiv \frac{\sqrt{2}}{\sqrt{r}}-1,\;P_{-}=\frac{1}{P_{+}},\;P_{+}P_{-}=1,\\ & \left(P_{\pm }=1\;r=\frac{1}{2}\right),\;\lim_{r\to 0}P_{\pm }=\left(\begin{array}{c} +\infty \\ 0 \end{array}\right),\;\lim_{r\to 2}P_{\pm }=\left(\begin{array}{c} 0\\ +\infty \end{array}\right), \end{array}$$

where we adopted a shortened notation for the limits of the horizons $P_{\pm }$ of Equation (4). In the LBHs *P*-parametrization, we note the intersection of $P_{-}$ curve as “inner horizon” and $P_{+}$ as “outer horizon” and the values r=1/2, r=2, and the limiting r=0 correspondent to the singularity in the Schwarzschild limit. A schematic representation of the extended plane is rendered in the right panel, enlightening the BH regions (where $P_{+}=P_{-}^{-1})$. The LQG extended plane shows limiting points $P_{+}=0$ for r=0, and $P_{+}$ for r=1/2 (note the analogy with the maximum of the horizon curve of the Kerr geometries for a=M correspondent to the extreme Kerr BH). The limiting values $P_{\pm }=1/2$ are clear; this is an extreme point where $r_{+}=r_{-}$. To complete the analysis of plane in terms of the horizons $r_{\pm }$, we can consider the curves $P_{s}^{\pm }$ of the extended plane:

(5) $$\begin{array}{c} P_{s}^{\pm }\equiv \frac{1-r\pm \sqrt{1-2r}}{r}:\;r=r_{*}^{\pm }, \end{array}$$

where $P_{s}^{+}P_{s}^{-}=1$. Alternately, we consider the horizons in the ϵ−r plane, making explicit the dependence of *P* in terms of ϵ-loop parameter having the horizon:

(6) $$\begin{array}{c} \epsilon_{\pm }\equiv \frac{\sqrt{-4r^{2}+6r+2\sqrt{2}\sqrt{r}}}{\sqrt{{\left(1-2r\right)}^{2}}},\;\lim_{r\to ℘}\epsilon_{\pm }=0,\;℘\in \left\{0,2\right\}, \end{array}$$

(7) $$\begin{array}{c} \lim_{r\to 1/2}\epsilon_{\pm }=+\infty ,\;\epsilon_{s}\equiv \frac{\sqrt{r}}{\sqrt{\frac{1}{2}-r}}:\;P_{s}^{\pm }\left(\epsilon \right)=P\left(\epsilon \right). \end{array}$$

In $\epsilon_{s}$ of Equation (6), we consider Equation (5). The choice of the ϵ–representation has several advantages, evident from the horizons representations in the ϵ−r extended plane. The two limiting points r=0 and r=2 also are connected to the value ϵ=0, and the limit r=1/2 is a vertical asymptote for ϵ→+∞. On the other hand, there is $\partial_{\epsilon }^{2}\;r_{*}\left(\epsilon \right)=0$ on $\epsilon_{\pm }=1/\sqrt{3}$ for $r_{*}=1/8$—Figure 2. It is clear from Figure 2 $\epsilon_{s}$ is bounded in the range r∈[0,1/2].

We stress that, in the metric bundles analysis and the geometry properties distinguished with MBs, we are concerned with the properties an observer could measure in the region outside the outer horizon $r_{+}$; in this context, therefore, we consider observers at infinity, adopting an adapted frame. This obviously compels a reinterpretation of the metric bundles in the region close to the horizons $r_{\pm }$—see Figure 4. In this framework, the horizons confinement in the MBs sense may be interpreted as the presence of a “local causal ball” in the extended plane, which is a region where MBs are entirely confined in—i.e., no horizons’ replicas can be found in the other regions of the extended plane.

**Figure 5:**
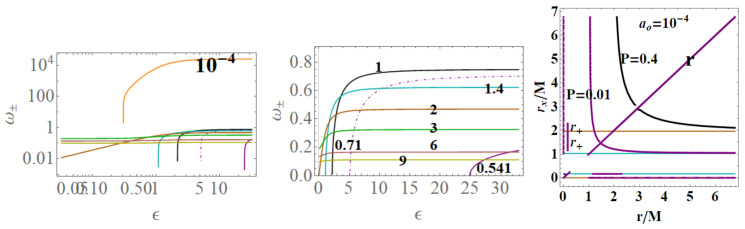
Left and center panels show the vertical lines of the extended plane; in other words, the MB intersections with the curves r= constant for different values of *r* signed in pictures, exploring different regions of the $\omega_{\pm }$ values of limiting photon orbital frequency. Further notes on notation are in Table 1. The right panel shows the solutions of the problem $\omega \left(r_{x}\right)=\omega_{\pm }\left(r\right)$ (“horizons”’ replicas in this spherically symmetric geometry); in other words, the horizontal lines in the extended plane for different polymeric metric parameters *P* and for a selected LQG area parameter $a_{o}$ (*M* is the ADM mass and $r_{\pm }$ are the BH horizons).

Figure 5 represent location of orbits $r<r_{x}$ at equal frequency ω (therefore belonging to the same bundle) in two cases. In the first case, we consider the same geometry, with $P=P_{x}$ corresponding to a horizontal line of the extended plane (implying clearly certain conditions on the MBs curvature in the P−r plane). Clearly, we look particularly to the couple of radii *r* and $r_{x}$ such that the two radii are in an inner region of the extended plane, upper bounded of the horizon $P_{-}$, and the second outside the region bounded by the horizon curve $P_{+}$.

**Figure 6:**
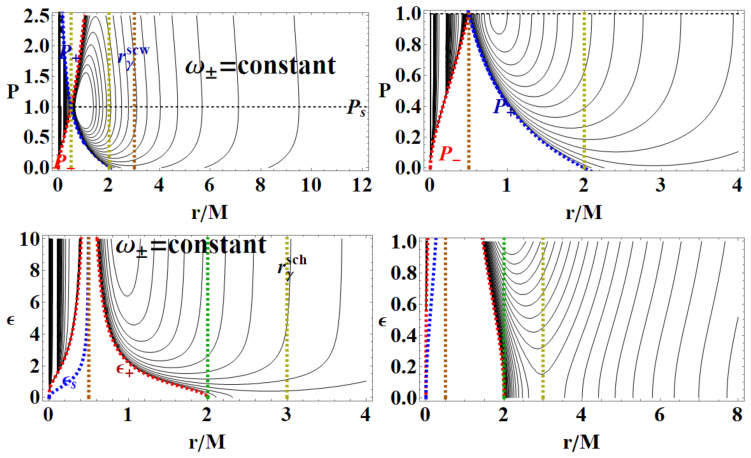
Metric bundles (MBs) in the extended plane P−r (upper panels) and ϵ−r (bottom panels) for ADM mass M=1. (*P* and ϵ are polymeric parameters). MB curves are the bundles at equal limiting photon orbital frequency $\omega_{\pm }$. Radius r=0.5 is relevant for the analysis of the horizons and r=2 is the horizon in the Schwarzschild limit, r=3 is the photon circular orbit in the Schwarzschild limit (a geodesic in this spacetime). Curves $P_{\pm }$ of the horizons, $P_{s}=P_{+}P_{-}=1$ is also shown. The Schwarzschild limit is for $P_{-}=0$. We note the presence of curves at r<2 for very small *P*; the role of r=3M is the photon orbit in the Schwarzschild limit (*M* is the Schwarzschild ADM mass). In the bottom panels, MBs are also shown in the plane ϵ−r, red curves are the horizons $\epsilon_{\pm }$, curve $\epsilon_{s}:r_{*}=r$ is also shown. Further notes on notation are in Table 1.

Detecting self-intersections of the bundles curves on the extended plane, in the same geometry (horizon confinement) or intersection of bundles curves in different geometries, is a crucial point in the MBs analysis, related to the issue of confinement and causal balls. There are no MB curves’ self-intersections, on equal *P* and *r* (a part some special cases such as extreme Kerr and RN BH spacetime); in other words, there are no MB knots. Figure 4 show on the other hand an analysis that is particularly interesting for the metric bundle approach where the curve r(P), a solution of the problem $g_{tt}=c_{t}=$ constant *and* $g_{rr}=c_{r}=$ constant, is showed. This curve collects the metric solutions (in terms of *P* parameter) having equal $g_{tt}$ and $g_{rr}$, on the same radius *r*; clearly, we are interested particularly to consider the values of *P* close to the maximum P=1 or for P<1. Figure 5 and Figure 6 show the metric bundles and vertical and horizontal lines in the P−r and ϵ−r extended plane. The approximations of the bundles to the horizons curves and the study of horizons’ replicas for different values of the parameter are clear. In Section 3, we also consider the explicit expression of the metric bundles, as solutions $L_{N}=0$, adopting different parameterizations.

### 2.2. Comparison with the Reissner–Norström Geometry

The Reissner–Norström (RN) metric is a well-known spherically symmetric (and static) electro-vacuum solution of Einstein equations, with inner $r_{-}$ and outer $r_{+}$ Killing horizons (with Killing vector $\xi_{t}$):

(8) $$\begin{array}{c} r_{-}\equiv M-\sqrt{M^{2}-Q^{2}};\;r_{+}\equiv M+\sqrt{M^{2}-Q^{2}}; \end{array}$$

(here, we adopt usual spherical symmetric coordinates (t,r,θ,ϕ), where also $\sigma \equiv \sin^{2}\theta$). There is a naked singularity for Q>M, the extreme RN-BH geometry, where $r_{\pm }=M$ occurs in the limit Q=M. We construct the metric bundles $Q_{\omega }$, introducing the Killing field L and the zero-quantity $L_{N}$ as follows:

(9) $$\begin{array}{cc} & L\equiv \xi_{t}+\omega \xi_{\phi };\;L_{N}\equiv g\left(L,L\right)=g_{tt}+g_{\phi \phi }\omega^{2}=\sqrt{r}\sqrt{r^{3}\sigma \omega^{2}-r+2}=0, \end{array}$$

(10) $$\begin{array}{c} \omega_{\pm }=\pm \frac{-g_{tt}}{g_{\phi \phi }}=\pm \frac{\sqrt{Q^{2}+\left(r-2\right)r}}{r^{2}},\;Q_{\omega }\equiv \sqrt{r}\sqrt{r^{3}\sigma \omega^{2}-r+2}, \end{array}$$

(11) $$\begin{array}{c} Q_{\omega }=0,\;\mathrm{for}\;\omega =\omega_{Sch}\equiv \frac{\sqrt{r-2}}{r^{3/2}},\;or\;r=0 \end{array}$$

$\omega_{Sch}$ is the frequency $\omega_{\pm }$ in the Schwarzschild geometry. In the RN geometry, to make it easier to read, we use geometric units where M=1 in many quantities. Here, and in the following, since the metric is spherically symmetric, we can use, where more convenient, σ=1, i.e., we fix an arbitrary equatorial plane without loss of generality. On the other hand, the re-parametrization $\sigma \omega^{2}\to \omega^{2}$ is an important definition adopted also in the axially symmetric case of the Kerr MBs.

**Figure 7:**
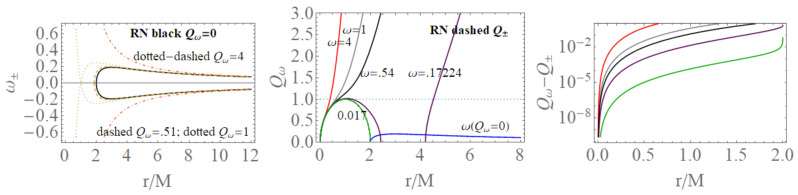
Reissner–Norström (RN) analysis: $Q_{\omega }$ are the metric bundles of Equation (9), ω is the bundle frequencies, $Q_{\pm }$ is the horizon curve in the extended plane, frequency solution of $Q_{\omega }=0$ is in Equation (11). (Here, *M* is the metric mass parameter of the Reissner–Norström line element). The right panel is the difference $Q_{\omega }-Q_{\pm }$ versus r/M, for the frequency values as in the central panel. The left panel shows the frequencies $\omega_{\pm }$ of Equation (9) versus r/M as function of different *Q* from BH to NS (naked singularities)—see also Table 1.

Functions $\omega_{\pm }$ are limiting light-like particles orbital frequencies showed in Figure 9; they constitute the limiting conditions for the measure of the stationary light-like observing orbital frequencies. The RN horizon $Q_{\pm }=\pm \sqrt{r(2-r)}$ in the extended plane has a similar form to the Kerr geometry horizon $a_{\pm }=\pm \sqrt{r(2-r)}$—see Figure 2—where $\Upsilon \in \{|Q_{\pm }|,|a_{\pm }|\}$ has values in [0,M], where Υ=0 corresponds to the Schwarzschild geometry and Υ=M is for the extreme RN (Q=M) or extreme Kerr BH case, respectively. For both geometries, this is a maximum of the horizon curve in the extended plane corresponding to the extreme BH solution. The horizon curve is closed and bounded in the range r∈[0,2M], where r=0 is the Schwarzschild, Kerr, or RN central singularity, r=2M is the horizon in the Schwarzschild limit of the RN and Kerr geometry as well as in the LBHs. The analysis of the RN MBs in Figure 7 shows that it is $Q_{\pm }=Q_{\omega }$ only for σ=0 or ω=0, or r=0. In fact, the absence of a tangency condition of the bundles with the horizon curves is obviously an expression of the spherical symmetry. On the other hand, the approaching of MB curves to the horizons and the role of electric charges are shown in Figure 7. There is also ω=0 only for bundles confined in the region r≤2. The choice of bundle parametrization for the RN case is clear, being related to the horizons’ definitions. The MB introduction in the RN geometries clarifies some aspects of the NSs, and aspects of (local) causal structure on the bases of the horizons properties, and individuates the bottleneck region typical of certain NSs. The precise definition of bottleneck goes far from the goals of the present analysis of the regular LBH solutions; however, the bottleneck is a restriction of the surfaces defined by the functions $\omega_{\pm }$ in the plane r−ω, as a frequency tunnel (or equivalently the light-surfaces (LSs)) for some solutions of NSs close, in the extended plane, to the extreme BH solution. The right panel of Figure 7 shows the approximation of the bundles to the horizon curves in the extended plane typical of a spherically symmetric case. It is clear that the distance between the MBs and the horizons’ curve decreases with the characteristic frequencies and increases with r∈[0,2M]. The bundles zeros curve in the central panel ω(Q=0) is the limiting Schwarzschild frequencies. The left panel shows the frequencies $\omega_{\pm }\left(r\right)$ (or the LSs in the plane r−ω) making evident the symmetries for negative and positive frequency values (in the case of spinning BHs, this symmetry with respect to positive and negative frequencies is broken). Increasing the value of the electric charge *Q* from the Schwarzschild Q=0 to NSs Q>M, the frequency curves are very different from the BH with the self intersecting curve of the extreme RN–BH, which is, however, a regular maximum point of the horizon curve in the extended plane. The self-intersections of the MBs is in fact a relevant aspect of the MB features.

## 3. Metric Killing Bundles of the LBHs

The four-velocity of the stationary observers are adapted to the Killing field $L=\xi_{t}+\omega \xi_{\phi }$ in the limiting Schwarzschild geometry and considered here in metric (1). We consider the null-like condition on the norm $L_{N}$

(12) $$\begin{array}{c} L_{N}=g\left(L,L\right)=\frac{\sigma \omega^{2}\left(a_{o}^{2}+r^{4}\right)}{r^{2}}-\frac{\left(r-2m\right){\left(2mP+r\right)}^{2}\left(r-2mP^{2}\right)}{a_{o}^{2}+r^{4}}=0. \end{array}$$

It is obvious that $L_{N}$ is zero on the horizons only for ω=0 ($g(\xi_{t},\xi_{t})=0$—static observers are defined by the particular solution ω=0; these observers, for example, cannot exist in the ergoregion of a Kerr geometry). Note that we can use also an adapted parametrization as

(13) $$\begin{array}{c} L_{N}=\frac{\left(R-2\right)\left(R-2P^{2}\right){\left(2P+R\right)}^{2}}{A_{o}^{2}+R^{4}}+\frac{W^{2}\left(A_{o}^{2}+R^{4}\right)}{R^{2}}; \end{array}$$

(14) $$\begin{array}{c} \mathrm{where}\;\{r\to mR,a_{o}\to A_{o}m^{2},\omega \to W/\left(m\sqrt{\sigma }\right)\}. \end{array}$$

In the plane (P,R), the outer horizon is $P_{+}\equiv \sqrt{1/2R}$. (We should also note that, in this way, *W*, *R*, and the length $A_{o}$ depend on the ADM mass *M* and the polymeric function *P*. The frequency $\omega \sqrt{\sigma }$ re-parametrization is instead a typical property of the MBs, which is generally related, for static as well as axially symmetric solutions to the MBs origin r=0 properties).

We can explore the spherical symmetry in the context of the metric bundles; it is clear that we can take advantage of this symmetries by considering σ=1, i.e., an (Schwarzschild BH) equatorial plane, without loss of generality. However, on $r_{\pm }$ and $r_{s}$, there is

(15) $$\begin{array}{c} \;L_{N}\left(r_{+}\right)=\frac{\sigma \omega^{2}\left(a_{o}^{2}+16m^{4}\right)}{4m^{2}}, \end{array}$$

(16) $$\begin{array}{c} \;L_{N}\left(r_{-}\right)=\frac{\sigma \omega^{2}\left(a_{o}^{2}+16m^{4}P^{8}\right)}{4m^{2}P^{4}}, \end{array}$$

(17) $$\begin{array}{c} L_{N}\left(r_{*}\right)=\frac{64m^{4}{\left(P-1\right)}^{2}P^{3}}{a_{o}^{2}+16m^{4}P^{4}}+\frac{\sigma \omega^{2}\left(a_{o}^{2}+16m^{4}P^{4}\right)}{4m^{2}P^{2}}. \end{array}$$

Therefore, more precisely:

(18) $$\begin{array}{c} \left(r_{-}\right):\;L_{N}\left(r_{-}\right)=0,\;\sigma =0,\;\omega >0,\;P\in ]0,1],\;\omega =0,\;P\in ]0,1], \end{array}$$

(19) $$\begin{array}{c} \left(r_{+}\right):\;L_{N}\left(r_{+}\right)=0,\;\sigma =0,\;\omega >0,\;P\in \left[0,1\right],\;\omega =0,\;P\in \left[0,1\right], \end{array}$$

(20) $$\begin{array}{c} \;\left(r_{*}\right):\;L_{N}\left(r_{*}\right)=0,\;\sigma =0,\;\omega >0,\;P=1,\;\omega =0,\;P=1. \end{array}$$

### 3.1. Light Surfaces (LS) Frequencies

Adopting the procedure discussed in Section 2.2, we evaluate the zero-quantity $L_{N}$, obtaining the light-like orbital frequencies $\omega_{\pm }$

(21) $$\begin{array}{cc} & L_{N}=0,\;\omega_{\pm }\left(m,P\right)\equiv \pm \frac{-g_{tt}}{g_{\phi \phi }}=\pm \frac{G(r)}{H(r)\sigma }=\pm \frac{\sqrt{\frac{\left(2m-r\right)\left(2mP^{2}-r\right){\left(2mP+r\right)}^{2}}{a_{o}^{2}+r^{4}}}}{\sqrt{\frac{\sigma \left(a_{o}^{2}+r^{4}\right)}{r^{2}}}}, \end{array}$$

(22) $$\begin{array}{c} \;\omega_{\pm }\left(M,P\right)=\pm \frac{\sqrt{\frac{\left(2M-{\left(P+1\right)}^{2}r\right)\left(2MP^{2}-{\left(P+1\right)}^{2}r\right){\left(2MP+{\left(P+1\right)}^{2}r\right)}^{2}}{{\left(P+1\right)}^{8}\left(a_{o}^{2}+r^{4}\right)}}}{\sqrt{\frac{\sigma \left(a_{o}^{2}+r^{4}\right)}{r^{2}}}}, \end{array}$$

which are also the characteristic frequencies of the bundles. Note that these frequencies also define the light surfaces in many aspects of BH physics and more generally in the processes of energy extractions. In these spherically symmetric geometries, consider the magnitude $|\omega_{\pm }|$. The “Schwarzschild” limit is:

(23) $$\begin{array}{c} \omega_{Sch}\equiv \pm \frac{\sqrt{-\frac{2M-r}{r}}}{\sqrt{r^{2}\sigma }},\\ \lim_{a_{o}\to 0}\lim_{P\to 0}\omega_{\pm }\left(x\right)=\omega_{Sch},\;x=\left\{\left(m,P\right),\left(M,P\right)\right\}. \end{array}$$

**Figure 8:**
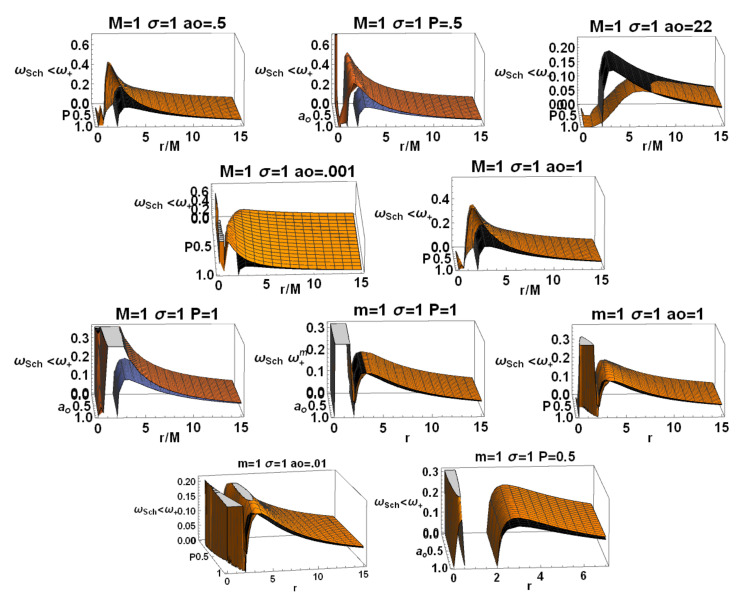
Limiting light-like frequencies $\omega_{\pm }\left(x\right)$ (orange) with x={m,M} (*M* is the ADM mass, *m* is the polymeric mass), solutions of $L_{N}=0$ and $\omega_{Sch}$ the light-like frequencies of the Schwarzschild geometries, as functions of *r*, the LQG area parameter $a_{o}$, and the metric polymeric *P* and different values of *P*, and $a_{o}$, respectively. There is $\sigma \equiv \sin^{2}\theta$, where σ=1 is the Schwarzschild BH equatorial plane—see Equation (21) and Table 1.

The two frequencies $\omega_{\pm }\left(x\right)$ and $\omega_{Sch}$ coincide for special values of $a_{o}$ and *P*: there is generally $\omega_{\pm }\left(x\right)\geq \omega_{Sch}$ with exceptions we show in Figure 8. On the other hand, these figures show differences between $\omega_{\pm }\left(x\right)$ considering as functions of (m,P) or (M,P). Clearly at the horizons, there is $\omega_{\pm }\left(r_{\pm }\right)=0$ and $\lim_{r\to 0}\omega_{\pm }=0.$

The significance of these frequencies relies on the fact that they can be connected directly with the observations of properties that are also conformal invariants determining several properties of the geometry. We find therefore the limiting frequencies that have an extreme for the loop mass *m*, which is

(24) $$\begin{array}{c} \;m_{\tau }:\;\partial_{m}\omega_{\pm }=0, \end{array}$$

(25) $$\begin{array}{c} m_{\tau }\equiv \frac{\sqrt{{\left(-6P^{2}+4P-6\right)}^{2}P^{2}r^{2}+64\left(P^{2}-2P+1\right)P^{3}r^{2}}-P\left(-6P^{2}+4P-6\right)r}{32P^{3}} \end{array}$$

**Figure 9:**
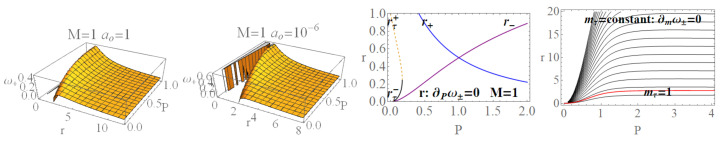
3D plots show the limiting frequencies $\omega_{\pm }$ of stationary observers in the spacetime (26) for light surfaces as a function of *r* and *P* (polymeric metric parameter) for different LQG length (area) parameter $a_{o}$, frequencies are in Equation (9). The extremes as function of *P* are shown in the 2D third and fourth panels. Third panel: radii $r_{\tau }^{\pm }$ of Equation (26) as functions of *P* for the ADM mass M=1 ($r_{\pm }$ are the BH horizons). Fourth panel: LQG mass parameter $m_{\tau }=$ constant of Equation (24) in the plane (r,P), see also Table 1.

Figure 9. More precisely, limiting photon orbital frequencies have an extreme for the polymer function, *P* different according to the different orbit *r*,

(26) $$\begin{array}{c} r_{\tau }:\;\partial_{P}\omega_{\pm }=0\;\left(M=1\right)\\ r_{\tau }^{\pm }\equiv \frac{1}{2}\left(\pm \sqrt{\frac{8}{{\left(P+1\right)}^{2}}-\frac{8}{P+1}+1}-\frac{4}{P+1}+\frac{4}{{\left(P+1\right)}^{2}}+1\right). \end{array}$$

These radii are represented in Figure 9 with respect to the horizons.

The bundles are therefore a set of geometries, as defined by a leading parameter of the chosen parametrization such that all the geometries of the bundles are only those characterized by a photon limiting orbital frequency ω, the bundle characteristics’ frequency is in the Kerr applications, and the BH horizon frequency in the extended plane. In the spherical symmetric spacetime, we investigate in this work the frequencies connecting geometries very close to the horizon (in the extended plane) with other geometries. Mainly here we consider the *P* parametrization. Alternately, we can consider the ϵ-parametrization in the extended plane of Figure 2. The horizons’ curves are the functions $P_{\pm }$ of Equation (4). Clearly, we could have used the $\epsilon_{\pm }$ representation in a plane ϵ−r as in Figure 2. The vertical lines in the extended plane r= constant represent a fixed point in different geometries; the collections of all points on the bundles at r= constant provide the set of different or equal frequencies ω connecting therefore different solutions making evident the modifications of the frequencies due to the shift of the polymeric functions. The horizonal lines in the extended plane, correspondent to P= constant, represent one geometry with P= constant, and the crossing of the horizontal line with all the bundles of the plane provides the set of light surfaces r±(ω) solutions of $L_{N}=0$.

### 3.2. Metric Bundles Parametrization

In this section, we provide an explicit expression for MBs according to different parameterizations.

**Figure 10:**
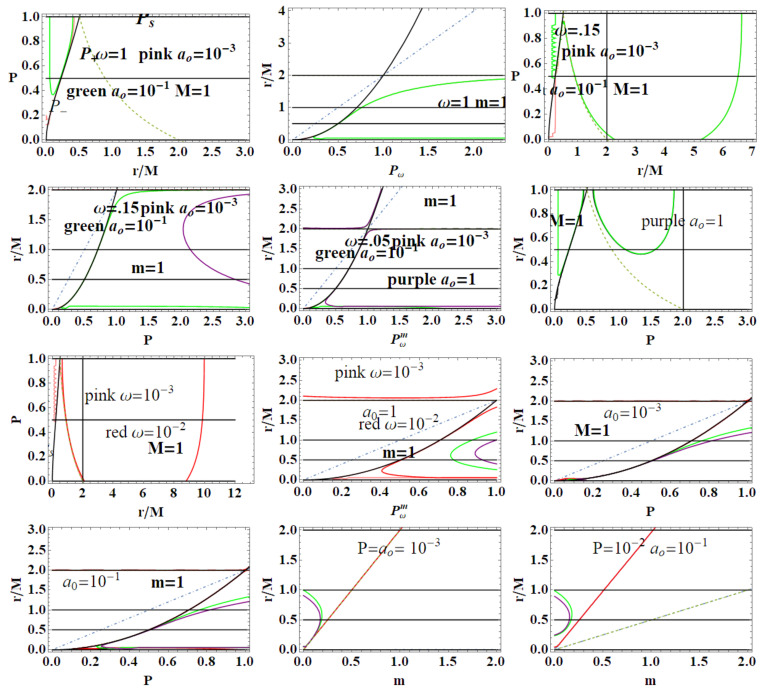
Metric bundles (solutions of $L_{N}=0$, connected to the light surfaces) for selected values of the polymeric model parameters as functions of the polymeric parameter *P* or loop mass *m* (*M* is the ADM mass parameter) in the plane (r,P) or (r,m) for different bundle frequencies ω (according to colors reported in panels). $a_{o}$ is the LQG length parameters. Horizons $r_{\pm }$ and radius $r_{*}$ are also shown—see also Table 1 for details on the notation.

**Figure 11:**
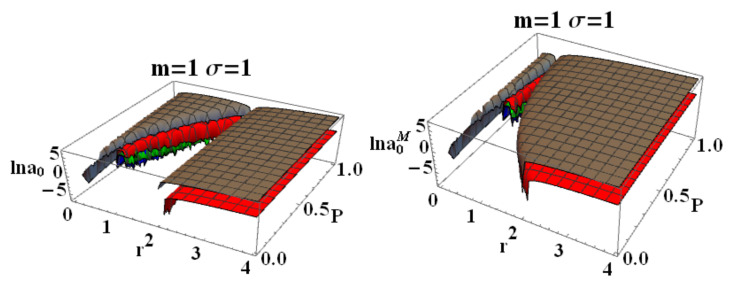
3D plots represent metric bundles $a_{o}^{\pm }\left(m,P\right)$ for $a_{o}$-parametrization see Equation (28), for $\omega =10^{-4}$ (gray) ω=0.1 (red), ω=1 (green), where $a_{o}$ is the LQG length parameters, *P* is the polymeric metric parameter, ω is the light-like orbital limiting frequencies (stationary observers), *M* is the ADM mass, *m* is a polymeric mass, only asymptotically equivalent to the ADM mass. In the panel, we adopt the notation $a_{o}=a_{o}^{\pm }\left(m,P\right))$ and $a_{o}^{M}=a_{o}^{\pm }\left(M,P\right)$ (right panel). We also took advantage of the symmetries $a_{o}^{\pm }=\mp a_{o}^{\mp }$, and $\sigma \equiv \sin^{2}\theta$, the (BH Schwarzschild) equatorial plane is σ=1—see also Table 1 for details on the notation.

- **Metric bundles: parametrization according to σ** As the metric is spherically symmetric, we can consider, without loss of generality, σ=1, i.e., a fixed (Schwarzschild BH) equatorial plane. Nevertheless, we could consider explicitly a parametrization according to the “poloidal” angle θ, obtaining the curves:
  (27) $$\begin{array}{cc} & \sigma_{\omega }=\frac{r^{2}\left(r-2m\right){\left(2mP+r\right)}^{2}\left(r-2mP^{2}\right)}{\omega^{2}{\left(a_{o}^{2}+r^{4}\right)}^{2}}. \end{array}$$
  In here, metric bundles with ω= constant are on the hyperplane $\sigma_{\omega }=1$. Clearly, this choice is relevant for the parametrization $W=\omega \sqrt{\sigma }$. Note that there is $\sigma_{\omega }=0$ for $r\in \{0,r_{\pm }\}$. Figure 10 represent special and limiting cases of metric bundles.
- **Metric bundles: $a_{o}$-parametrization**
  It is relevant to study a $a_{o}$-parametrization to consider the families of metrics for different minimum areas parameter $a_{o}$. Implementing therefore the notion of metric bundles with the area parameter, we obtain explicitly
  (28) $$\begin{array}{c} {\left(a_{o}^{\pm }\left(m,P\right)\right)}^{2}\equiv \pm \sqrt{\pm \frac{\sqrt{r^{2}\sigma \omega^{2}\left(r-2m\right){\left(2mP+r\right)}^{2}\left(r-2mP^{2}\right)}}{\sigma \omega^{2}}}-r^{4},\\ {\left(a_{o}^{\pm }\left(M,P\right)\right)}^{2}\equiv \frac{\sqrt{{\left(P+1\right)}^{8}r^{2}\omega^{2}\left(P^{2}\left(r-2\right)+2Pr+r\right)\left({\left(P+1\right)}^{2}r-2\right){\left({\left(P+1\right)}^{2}r+2P\right)}^{2}}}{{\left(P+1\right)}^{8}\omega^{2}}-r^{4} \end{array}$$
  represented in Figure 11. Providing constraints on the loop minimal areas, functions $\left(a_{o}^{\pm }\left(M,P\right)\right),a_{o}^{\pm }\left(m,P\right)))$ are not well defined on the horizons in the extended plane, and limits to r=0 is null.
- **Metric bundles: *P*-parametrization**
  Here, we consider the leading parameter *P*. Metric bundles in the extended plane P−r and ϵ−r are shown in Figure 6, where there is also a focus on the vertical and horizontal lines of the extended planes and horizons’ replicas at different values of the parameter.
  The equation for the metric bundles according to the *P*-parametrization (M=1) is polynomial function of degree 8, $f\left(P;\zeta \right)=\sum_{i=0}^{8}P^{i}\zeta_{i}$, where
  (29) $$\begin{array}{c} \zeta_{0}\equiv \omega^{2}{\left(a_{o}^{2}+r^{4}\right)}^{2}-\left(r-2\right)r^{5};\\ \zeta_{1}\equiv 8\omega^{2}{\left(a_{o}^{2}+r^{4}\right)}^{2}+8\left(-r^{2}+r+1\right)r^{4};\\ \zeta_{2}\equiv 4\left[7\omega^{2}{\left(a_{o}^{2}+r^{4}\right)}^{2}+\left[r\left(r\left(2-7r\right)+6\right)+2\right]r^{3}\right];\\ \zeta_{3}\equiv 8\left[7\omega^{2}{\left(a_{o}^{2}+r^{4}\right)}^{2}-r^{4}\left(7r^{2}+r-3\right)\right];\\ \zeta_{4}\equiv 2\left[35\omega^{2}{\left(a_{o}^{2}+r^{4}\right)}^{2}-r^{2}\left[r\left(r\left[5r\left(7r+2\right)-8\right]+8\right)+8\right]\right];\\ \zeta_{5}\equiv 8\left[7\omega^{2}{\left(a_{o}^{2}+r^{4}\right)}^{2}-r^{4}\left(7r^{2}+r-3\right)\right];\\ \zeta_{6}\equiv 4\left[7\omega^{2}{\left(a_{o}^{2}+r^{4}\right)}^{2}+\left[r\left(r\left(2-7r\right)+6\right)+2\right]r^{3}\right];\\ \zeta_{7}\equiv 8\left[\omega^{2}{\left(a_{o}^{2}+r^{4}\right)}^{2}-r^{6}+r^{5}+r^{4}\right]; \end{array}$$
  (30) $$\begin{array}{c} \;\zeta_{8}\equiv \omega^{2}{\left(a_{o}^{2}+r^{4}\right)}^{2}-\left(r-2\right)r^{5}. \end{array}$$

## 4. The LBHs Thermodynamical Properties

In this section, we review some aspects of the BH thermodynamics. In Section 4.1, we explore the BHs surfaces gravity, the luminosity, and the temperature in terms of the loop model parameters; then, these quantities are considered on metric bundles. We discuss connections between different geometries of one bundle considering their thermodynamical properties–[11]. In general, the Hawking emission at $r_{+}$ leads to the evaporation process with BH Bekenstein–Hawking temperature. The mass loss on the process can be deduced with the luminosity (*L*). The focusing idea is how the BH thermodynamical properties can explain and distinguish the construction of the underling graph in the LQG model adopted here, especially on the grounds of the MBs structures analyzing the horizons properties in regions far from the horizons—see also [1,31].

### 4.1. BHs Thermodynamics and LQG Parameters

In this section, we evaluate the BH areas and the BHs surfaces gravity and temperature in terms of the LBH parameters P.

**Figure 12:**
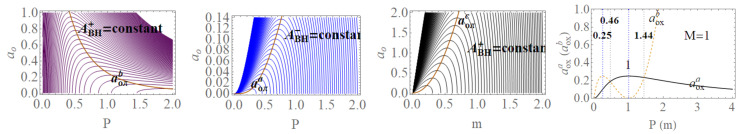
Curves of constant BH areas $A_{\mathrm{BH}}^{\pm }$ (BH areas relatives to BH horizons $r_{\pm }$) are shown. Left first and second panels: $A_{\mathrm{BH}}^{\pm }$ in the $\left(a_{o},P\right)$ plane, respectively. Third panel: area $A_{\mathrm{BH}}^{+}$ in the $\left(a_{o},m\right)$ plane. Details on the notation can be found in Table 1. Extreme length parameter $a_{o\pi }^{i}$ for i∈{a,b,c} is also shown—Equations (34) and (35). Here, $a_{o}=A_{min}/8\pi$ is an area parameter where $A_{min}$ is the minimum area gap of LQG, *P* is a metric polymeric parameter, and *M* is the ADM mass in the Schwarzschild limit, while *m* is a parameter depending on the polymeric function. Right panel: loop length curves $a_{ox}^{b}$ ($a_{ox}^{a}$) as function of the loop mass *m* (polymeric parameter *P* for M=1)—Equation (33). $a_{ox}^{b}$, $a_{ox}^{a}$ are solutions of $A_{\mathrm{BH}}^{-}=A_{\mathrm{BH}}^{+}$ for the BH areas.

**Figure 13:**
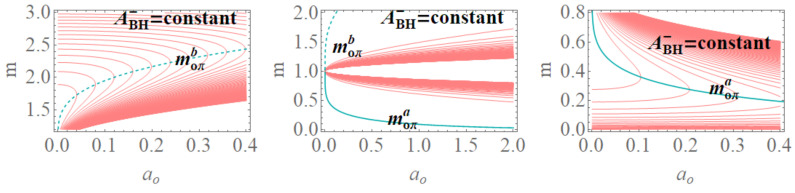
Curves of constant BH areas $A_{\mathrm{BH}}^{-}$ (BH area function on $r_{-}$) in $\left(m,a_{o}\right)$ the plane. Different panels show a focus on ranges of *m*, where *m* is a mass parameter depending on the polymeric function and the ADM mass while $a_{o}=A_{min}/8\pi$ is an area parameter and $A_{min}$ is minimum area appearing in LQG (minimum area gap of LQG). Extreme loop mass $m_{o\pi }^{a}$ and $m_{o\pi }^{b}$ curves are shown— Equations (34) and (35). Details on the notation can be found in Table 1.

**Figure 14:**
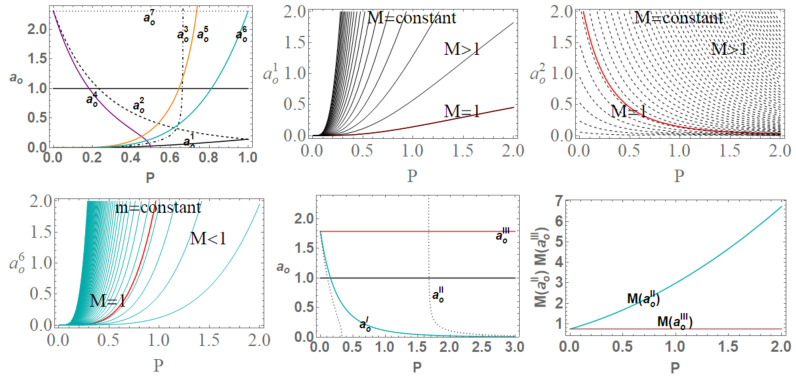
Left upper panel: quantities $a_{o}^{i}$ for i∈{1,…,7} of Table 2 as functions of the metric polymeric parameter P∈[0,1] for M=1 or m=1 (*M* is the ADM mass in the Schwarzschild limit and *m* is parameter depends on the polymeric function). Upper center and right panels and bottom-left panels show $a_{o}^{i}$ as functions of *P* for M= constant and m= constant. The center bottom panel shows $a_{o}^{\upsilon }$ for υ∈{I,II,III} of Equation (44) solutions of $\partial_{a_{o}}L\left(M,P\right)=0$, where $a_{o}=A_{min}/8\pi$, is an area parameter where $A_{min}$ is a minimum area appearing in LQG (minimum area gap of LQG). The bottom right panel represents $M(a_{o}^{i})=$ constant in the plane $\left(P,a_{o}\right)$. See also Table 1 for further details on the notation.

**Figure 15:**
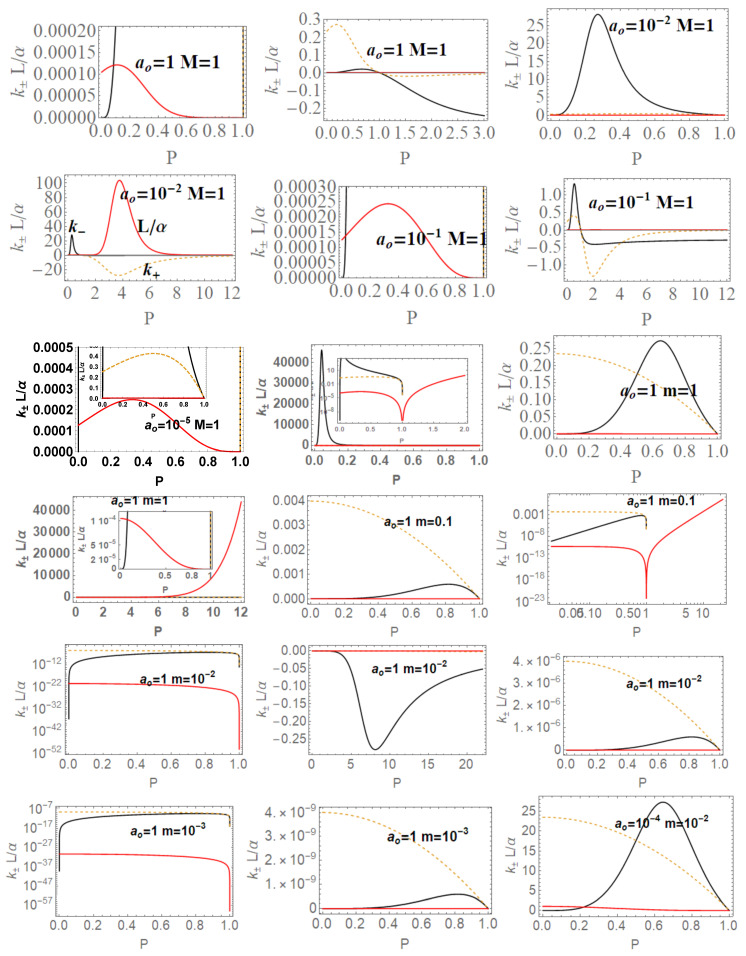
Plots of the surface gravity $\kappa_{\pm }$ and luminosity L/α as a function of the polymeric parameter, (α is a constant) evaluated in the two different approaches and for selected values of the parameters—see also Table 1. $a_{o}=A_{min}/8\pi$ is an area parameter where $A_{min}$ is a minimum area appearing in LQG (minimum area gap of LQG). *P* is the metric polymeric parameter, *M* is the ADM mass in the Schwarzschild limit, while *m* is a parameter that depends on the polymeric function.

**Figure 16:**
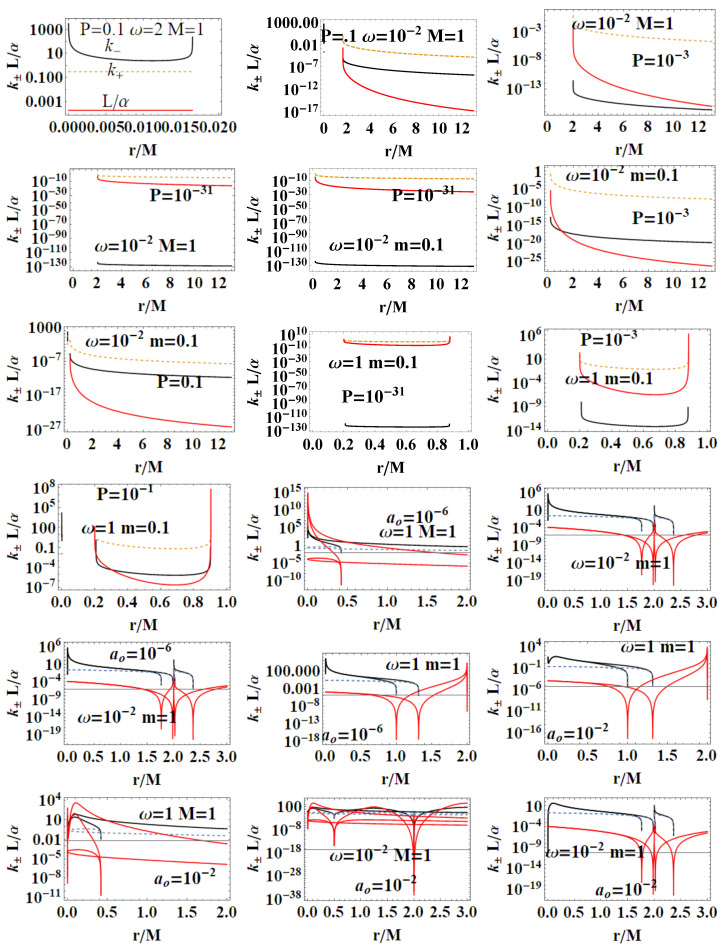
Plots of the surface gravity $\kappa_{\pm }$ and luminosity L/α, α is a constant as functions of *r* evaluated on the metric bundles of $a_{o}\left(\omega \right)$ or $P_{\omega }$ solution for metric bundles, for different values of the parameters as signed on the panel. Table 1 contains further details on the notation. $a_{o}=A_{min}/8\pi$ is an area parameter where $A_{min}$ is a minimum area appearing in LQG (minimum area gap of LQG). *P* is the metric polymeric parameter, *M* is the ADM mass in the Schwarzschild limit while *m* is a parameter that depends on the polymeric function, ω is the bundle (light-like orbital stationary frequency).

- **BH areas** We can evaluate the BH areas as follows:
  (31) $$\begin{array}{c} \left(A_{\mathrm{BH}}^{+}\right):\;A_{\mathrm{BH}}^{+}\left(m\right)=\frac{\pi \left(a_{o}^{2}+16m^{4}\right)}{m^{2}},\;\;A_{\mathrm{BH}}^{+}\left(M,P\right)=\pi \frac{{\left(P+1\right)}^{4}\left(a_{o}^{2}+\frac{16M^{4}}{{\left(P+1\right)}^{8}}\right)}{M^{2}}.\\ \left(A_{\mathrm{BH}}^{-}\right):\;A_{\mathrm{BH}}^{-}\left(m,P\right)=\pi \frac{\left(a_{o}^{2}+16m^{4}P^{8}\right)}{m^{2}P^{4}},\;A_{\mathrm{BH}}^{-}\left(M,P\right)=\pi \frac{{\left(P+1\right)}^{4}\left(a_{o}^{2}+\frac{16M^{4}P^{8}}{{\left(P+1\right)}^{8}}\right)}{M^{2}P^{4}}, \end{array}$$
  (32) $$\begin{array}{c} \;\;\;A_{\mathrm{BH}}^{-}\left(M,P\left(m\right)\right)=\pi \frac{\left(a_{o}^{2}+16{\left(\sqrt{m}-\sqrt{M}\right)}^{8}\right)}{{\left(\sqrt{m}-\sqrt{M}\right)}^{4}}. \end{array}$$
  In $A_{\mathrm{BH}}^{-}\left(M,P\left(m\right)\right)$, we used the quantity $P\left(m\right)=\frac{\sqrt{M}-\sqrt{m}}{\sqrt{m}}$, where $A_{\mathrm{BH}}^{\pm }$ is related to the surface bounded by the outer and the inner BH horizon. (In the extended plane, it is necessary to consider horizons $r_{\pm }$). Note that $A_{\mathrm{BH}}^{+}$ does not depend explicitly on *P*. When considered in the extended plane, there are some special values of the P parameters for which there is a coincidence of the areas $A_{\mathrm{BH}}^{\pm }$, for the equal values of $\left(m,a_{o}\right)$. There is $A_{\mathrm{BH}}^{-}\left(m,a_{o}\right)=A_{\mathrm{BH}}^{+}\left(m,a_{o}\right)$ (within the assumption M=1) for m=1/4 or $a_{o}=a_{ox}^{b}$, while $A_{\mathrm{BH}}^{-}\left(P,M\right)=A_{\mathrm{BH}}^{+}\left(P,M\right)$ for $a_{o}=0$, P=1 or $a_{o}=a_{ox}^{a}$, where
  (33) $$\begin{array}{c} a_{ox}^{a}\equiv \sqrt{\frac{16M^{4}P^{4}}{{\left(P+1\right)}^{8}}},\;a_{ox}^{b}\equiv \sqrt{16{\left(\sqrt{m}-1\right)}^{4}m^{2}}, \end{array}$$
  see Figure 12. Interestingly, however, the LBHs areas have extreme points: $\partial_{P}A_{\mathrm{BH}}^{-}=0$ for $a_{o}=a_{o\pi }^{a}$ and $\partial_{a_{o}}A_{\mathrm{BH}}^{\pm }=0$ for $a_{o}=0$; finally, $\partial_{P}A_{\mathrm{BH}}^{+}=0$ for $a_{o}=a_{o\pi }^{b}$, where
  (34) $$\begin{array}{cc} & a_{o\pi }^{a}\equiv 4\sqrt{\frac{P^{8}}{{\left(P+1\right)}^{8}}},\;a_{o\pi }^{b}\equiv \frac{4}{{\left(P+1\right)}^{4}}, \end{array}$$
  $\partial_{M}A_{\mathrm{BH}}^{-}=0$ for ($m_{o\pi }^{a},m_{o\pi }^{b})$, ($\partial_{a_{o}}A_{\mathrm{BH}}^{o}=0,\partial_{m}A_{\mathrm{BH}}^{o}=0$ for $a_{o}=0$), where
  (35) $$\begin{array}{cc} & m_{o\pi }^{a}\equiv \frac{1}{2}\left(-2\sqrt{2}\sqrt[{4}]{a_{o}}+\sqrt{a_{o}}+2\right),\;m_{o\pi }^{b}\equiv \sqrt{2}\sqrt[{4}]{a_{o}}+\frac{\sqrt{a_{o}}}{2}+1, \end{array}$$
  (M=1) see Figure 13, where the role of P=0.25 and P=1 is clear. Note, interestingly, the presence of an extreme of the BHs areas related to the graph loop parameters.
- **Surfaces gravity:** We can evaluate a LBH “surface gravity” correspondent to the outer and inner horizons $r_{\pm }$, respectively, as $\kappa_{\pm }\in \left\{\kappa_{\pm }\left(M,P\right),\kappa_{\pm }\left(m,P\right)\right\}$:
  (36) $$\begin{array}{c} \kappa_{-}:\;\kappa_{-}\left(m,P\right)=\frac{4m^{3}P^{4}\left(1-P^{2}\right)}{a_{o}^{2}+16m^{4}P^{8}},\;\kappa_{-}\left(M,P\right)=\frac{4M^{3}P^{4}\left(1-P^{2}\right)}{{\left(P+1\right)}^{6}\left(a_{o}^{2}+\frac{16M^{4}P^{8}}{{\left(P+1\right)}^{8}}\right)}; \end{array}$$
  (37) $$\begin{array}{c} \kappa_{+}:\;\kappa_{+}\left(m,P\right)=\frac{4m^{3}\left(1-P^{2}\right)}{a_{o}^{2}+16m^{4}},\;\kappa_{+}\left(M,P\right)=\frac{4M^{3}\left(1-P^{2}\right)}{{\left(P+1\right)}^{6}\left(a_{o}^{2}+\frac{16M^{4}}{{\left(P+1\right)}^{8}}\right)}. \end{array}$$
  Comparing the extended planes of Kerr geometries and the regular LBH geometries of Figure 2, we expect surface gravities to vanish in some extreme conditions on the loop graph parameters. It is then clear that $\kappa_{\pm }=0$ for P=1 and $\kappa_{\pm }>0$ for P<1, which is the region of the polymeric function values we explore here. Thus, there is
  (38) $$\begin{array}{c} \lim_{P\to 0}\kappa_{-}=0,\;\kappa_{+}\left(P\approx 0\right)=\frac{4m^{3}}{a_{o}^{2}+16m^{4}}-\frac{4m^{3}P^{2}}{a_{o}^{2}+16m^{4}}+O\left(P^{4}\right). \end{array}$$
  The limiting P=1, occurring in the extended plane of Figure 2 at r=1/2, is also an extreme for the $\kappa_{\pm }:\partial_{a_{o}}\kappa_{\pm }\left(M,P\right)=0$. Extremes $\partial_{M}\kappa_{\pm }\left(M,P\right)$ are for limiting conditions M=0, P=(0,1), $a_{o}=0$, relating the limiting values on P and the ADM mass. A further extreme is for minimal area $a_{o}=a_{o}^{1}$ (or $M=M(a_{o}^{1})$) for $\kappa_{-}\left(M,P\right)$ and $a_{o}=a_{o}^{2}$ for $\kappa_{+}\left(M,P\right)$. For convenience, we report in Table 2 all the relevant minimal areas $a_{o}^{i}$ for i∈{1,…,7}, introduced in this discussion and represented in Figure 14. Similarly, there is $\partial_{P}\kappa_{\pm }\left(M,P\right)=0$ for P=1 and M=0, and also for P∈[0,2/3] with $a_{o}=a_{o}^{3}$ for $\kappa_{-}\left(M,P\right)$ and P≤1/2 for $a_{o}=a_{o}^{4}$.
  There is an extreme for $\kappa_{\pm }\left(m,P\right)$ at $a_{o}\neq 0$ for the limiting condition M=0–([1]). There is then $\partial_{P}\kappa_{+}\left(m,P\right)$ for P=0, $\partial_{P}\kappa_{-}\left(m,P\right)=0$ for (m=0,P=0) and for $P\in ]0,\sqrt{2/3}[$ and $a_{o}=a_{o}^{5}$. On the other hand, $\partial_{m}\kappa_{-}\left(m,P\right)=0$, for the limiting cases m=0,P=(0,1) (including $\left(P=1a_{o}=0\right)$) and for $a=a_{o}^{6}$. Analogously, there is $\partial_{m}\kappa_{\pm }\left(m,P\right)=0$, an extreme condition having the special solution $a_{o}=a_{o}^{7}$, notably independent from the polymeric parameter.
  In Figure 14, we also considered an extended region of the P parameters.
- **The temperatures:** The evaluation of the temperature associated with the (regular) LBH proceeds directly in terms of surface gravity $\kappa_{+}$:
  (39) $$\begin{array}{c} T_{\mathrm{BH}}=\frac{\kappa_{+}}{2\pi }\;\mathrm{or}\;T_{\mathrm{BH}}\left(m\right)=\frac{{\left(2m\right)}^{3}\left(1-P^{2}\right)}{4\pi \left(a_{o}^{2}+{\left(2m\right)}^{4}\right)}. \end{array}$$
  We actually evaluate the temperatures $T_{\mathrm{BH}}^{\pm }$ in terms of $\kappa_{\pm }$, respectively, for the outer and inner horizons $r_{\pm }$. The interpretation and the evaluation of the temperature $T_{\mathrm{BH}}^{-}$ is debated in literature. In this analysis, while we intend clearly $T_{\mathrm{BH}}^{+}\equiv T_{\mathrm{BH}}$ as the BH temperature, when we intend the BH in the extended plane, as in Figure 15, then we need to consider $T_{\mathrm{BH}}^{\pm }$. On the other hand, considering the extended plane (P−r) or (ϵ−r), we expect the occurrence of an “extreme” case, where the temperature is null (similarly to the case of extreme Kerr BH) as made evident from the study of the surface gravity $\kappa_{\pm }$. Temperature is vanishing for m≈0, in the case $T_{\mathrm{BH}}^{+}\left(m,P\right)$:
  (40) $$\begin{array}{c} \;\lim_{m\to \upsilon }T_{\mathrm{BH}}=0,\;\upsilon \equiv \left\{\infty ,0\right\}. \end{array}$$
  (41) $$\begin{array}{l} T_{\mathrm{BH}}\left(m\to \infty \right)=\frac{1-P^{2}}{8\pi m}+O\left({\left(\frac{1}{m}\right)}^{2}\right),\\ T_{\mathrm{BH}}\left(P\approx 0\right)=\frac{2m^{3}}{\pi (a_{o}^{2}+16m^{4})}-\frac{2m^{3}P^{2}}{\pi a_{o}^{2}+16\pi m^{4}}+O\left(P^{4}\right). \end{array}$$
  The analysis in Figure 15 investigates extended parameter regions, where negative surface gravity is possible. (The negative $\kappa_{\pm }$ and therefore $T_{\mathrm{BH}}^{\pm }$ is a delicate and intriguing aspect of classical and loop quantum BHs, tightly connected to the white holes definition—[26,27,28]).
- **The luminosity:** In the analysis of luminosity, we consider [1]. The luminosity can be estimated by considering the Stefan–Boltzmann law as $L\left(m\right)=\alpha A_{\mathrm{BH}}\left(m\right)T_{\mathrm{BH}}^{4}\left(m\right)$, where $A_{\mathrm{BH}}$ is the BH area (on horizon $r_{+}$), and α is a factor depending on the evaluation model adapted for the luminosity. However, in this work, we mainly consider the quantity L/α. By assuming α= constant, we focus on the analysis of luminosity with the variation of the P parameters of the LQG graph and on the metric bundles. Studying L(m)/α (or L(M)/α), we investigate the regular BH mass evaporation process (the energy flux particularly where BH evaporation occurs through the Hawking emission in the proximity of the BH outer horizon $r_{+}$, with a temperature evaluated according to the Bekenstein–Hawking law and connected therefore to the surface gravity $\kappa_{+}$). We perform our investigation considering different values of P and on the geometries connected by the metric bundles. The luminosity is, therefore, in terms of *m*:
  (42) $$\begin{array}{c} L\left(m\right)=\alpha A_{\mathrm{BH}}\left(m\right)T_{\mathrm{BH}}{\left(m\right)}^{4}=\frac{16\alpha m^{10}{\left(1-P^{2}\right)}^{4}}{\pi^{3}{\left(a_{o}^{2}+16m^{4}\right)}^{3}}. \end{array}$$
  In the Schwarzschild limit, P→0 (correspondent to m→M), there is
  (43) $$\begin{array}{c} L=\frac{16\alpha m^{10}}{\pi^{3}{\left(a_{o}^{2}+16m^{4}\right)}^{3}}-\frac{64P^{2}\left(\alpha m^{10}\right)}{\pi^{3}{\left(a_{o}^{2}+16m^{4}\right)}^{3}}+O\left(P^{4}\right). \end{array}$$
  We should note that: L(m)=0 for P=1 or m=0 (in this special analysis, we consider *m* and *P* independent—for the limiting condition m=0 for the approximate geometry; see, for example, discussion in [1]). It is, however, relevant to consider the extremes of luminosity function *L* (related to the BH evaporation process for mass loss). Therefore, conveniently, we introduce here the following special values of the minimal length parameter $a_{o}$:
  (44) $$\begin{array}{c} a_{o}^{I}\equiv \frac{4\sqrt{5}M^{2}}{5{\left(P+1\right)}^{4}},\;a_{o}^{II}\equiv 4\sqrt{\frac{M^{4}\left(3P-1\right)}{{\left(P+1\right)}^{8}\left(3P-5\right)}},\;a_{o}^{III}\equiv \frac{4\sqrt{5}m^{2}}{5}, \end{array}$$
  represented in Figure 14. Therefore, there is $\partial_{m}L\left(m\right)=0$ for $a_{o}=a_{o}^{III}$ and for some limiting cases on P (for example, vertices of the LBHs triangle in Figure 2, i.e., limiting geometries for parameters values as studied in Section 2). Considering explicitly dependence on *P*, there is $\partial_{P}L\left(m\right)=0$ and $\partial_{a_{o}}L\left(m\right)=0$ for the limiting cases on the parameters P. We now focus on the situations when m=m(M,P). In this case, there is an extreme for the minimal area. (We include also the extremes $\partial_{M}L\left(M,P\right)=0$ in the limiting cases and for $a=a_{o}^{I}$). Then, there is $\partial_{P}L\left(M,P\right)=0$ in the limiting cases and P∈[0,1/3] for $a=a_{o}^{II}$, where $\partial_{a_{o}}L\left(M,P\right)=0$ only for the limiting cases. In Figure 15, we consider different limiting cases on the LBH model parameters $P=(P,m,a_{o})$ on the BHs quantities $\kappa_{\pm }$, L/α, and the temperature $T_{\mathrm{BH}}^{\pm }$, making evident the presence of extreme points and even negative values of temperature in extended regions of parameters.
- **LBHs thermodynamical properties and MBs:** We now consider the quantities of the regular LBHs geometries, $\kappa_{\pm }$ (surface gravity) and L/α (luminosity) evaluated on the metric bundles of the geometry. This analysis will connect different geometries of the same metric bundle through their thermodynamical properties. This treatment of the thermodynamical properties and LQG-BH will also characterize the role of the graph parameters in shaping different solutions. Eventually, this analysis connects the extended plane parameter variation with the transition from a LBH solution to another solution. In Figure 16, we note the presence of singularities and the behaviors at increasing distance from the r=0 (the bundles’ origins). A transformation from one solution of the bundle to another follows transformations of $\left(\kappa_{\pm },L/\alpha \right)$ on the curves evaluated on the bundles. This analysis explores the possibility of a transition from one solution of a metric family to another geometry of the same family, which, for example, can occur after interaction of the attractor with the surrounding matter environment in non-isolated BH systems, which is the general case in the most common astrophysical environments. This process would lead a BH from a point to another point of its extended plane representation. (This transition could also involve, of course, for some other diverse processes, a transition of the graph parameters). The relevant aspect of this analysis is that this transition must carry the system from one point to another in the extended plane *along* an MB curve. This means that the observer from the initial state will see a transition of the fixed frequency from a point $r_{1}$ to $r_{2}\neq r_{1}$ (in general, there are no fixed point along r= constant), where $r_{1}$ and $r_{2}$ are two points along the bundle uniquely identified by the detection of the fixed photon frequency. Vice versa, the observer will be able to recognize at the fixed point through the photon orbital frequency variation in the external region any geometry transition in the extended plane (regulated by thermodynamic laws). At fixed frequency, there is **always one and only one bundle; furthermore, a bundle curve does not in general self-cross–** there is an absence of knots. Therefore, we also test the hypothesis that the bundles, connecting the solutions uniquely through their characteristic frequencies and defining the associated light surfaces, could have a role in such transitions. Obviously, the thermodynamic onset provides in the new points of the plane a series of quantities as surface of gravity luminosity or temperature that have evolved on the bundles as shown in these analyses. Therefore, these results have to be compared with the correspondent analysis of MB curves. It should be also noted that, in Figure 16, we have fixed, depending on the parametrization of the bundles, different parameters and the frequency. (The functions associated with these quantities are generally well defined far from the horizons. In the analysis, we have taken advantage of this property to evaluate in the extended plane these quantities also on the horizon curves as clear from the analysis in Figure 16. Whatever the parameterizations adopted and the fixed parameters set, the horizon points of the extended plane clearly highlighted by the vertices of the correspondent triangle in the representation of the Figure 2 indicate signs of singularity for these quantities).

## 5. Discussion and Final Remarks

We review the steps of this analysis, discussing the main results and further developments. In Section 2, we found the metric bundles for the LQG metric approximation, for the geometries considered in Section 2.1. Results are shown in Figure 6 and more extensively analyzed in Section 3.2, within different parameterizations, discussing through these structures the main characteristics of the family of geometries and the characteristics of the horizons $r_{\pm }$, through the light surfaces associated with the MBs. We have also clarified some aspects of construction of the extended plane, within the application discussed here, the choice of metric bundles parametrization, and the first formulation of BH thermodynamics within the MB scenario in Section 4. We clarified aspects of MB definitions in the static and spherically symmetric spacetime; see discussion in Section 2.1.1 and Section 2.1.2. An extensive characterization is shown in Figure 1 and Figure 4, which leads to the construction of the extended plane for these solutions. We explored two representations of the extended plane for this LQG-BH solution in Figure 2 and Figure 3 and interpreted by comparing with the (stationary) Kerr geometry extended plane in Section 2.1.2. In this section, we also found the horizons’ replicas, showed in Figure 5 and Figure 6. These steps lead to the comparison of the LBH with the case of Reissner–Norström (RN) geometry in Section 2.2. The LQG geometry under consideration shares different similarities with RN metrics also from the MBs’ stand point. Thus, the second part of this investigation starts, where we analyzed the LBHs’ thermodynamical properties—Section 4. We characterized the thermodynamical properties of the LBH solutions in the extended plane for different parameters—see Figure 12 and Figure 13, and on the metric bundles in Figure 15 and Figure 16, showing the divergences from the reference GR solution. These quantities are evaluated on the horizon curves in the extended plane and on the MBs, relating different geometries on the bundles curves with the different values of luminosity, temperatures, or BH areas. Divergences with respect to the expected results considering the reference (asymptotically) GR solution are shown in Figure 8 with respect to the limiting light-like frequencies ω used as characteristic frequencies of the bundles. (The analysis of bundles in the extended plane compares intrinsically with the asymptotical solution which in the plane is contained as points in line P=0). In Figure 9, we show results of the analysis on the horizontal line of the bundles’ structures, correspondent to the light surfaces on a specific geometry, and this analysis points out very clearly the presence of non-monotone behaviors of the frequencies ω with dependence on the metric parameters *m*, depending also on the ADM mass (therefore eventually after a mass shift following the BH interaction with the matter environment, or, possibly due to a “transition”, the “graph state” may undertake from one value of its characteristic parameters to another). Similar behavior is shown with the presence of maxima and minima in the LBHs areas and temperatures (surfaces gravity) as in Figure 12 and Figure 13, and evaluated on the metric bundles in Figure 15 and Figure 16. A relevant aspect of this analysis is that the replicas relate a region close to the horizons virtually in the sense of Figure 5 and Figure 6 and a region far from the “central” BH where there is a copy of the frequency ω. We can measure the discrepancies on the light frequencies (and consequently the timelike frequencies) as measured in these regions expecting a GR solution. Notably, the analysis may be interpreted as a deformation of aspects of causal structure (in the sense of causal ball, for example) within the extended plane representation of Figure 6 or Figure 5, relating graph properties to BHs thermodynamics with MBs.

We summarize below results of the investigation with some comments and contextualization.

***1.*****Constraining LQG solutions.** One purpose of this analysis is to provide constraints to the LQG mini-super-space polymeric regular BHs of Equation (1) within the framework provided by metric Killing bundles. The special framework is therefore here firstly applied to LQG–BH and BH thermodynamics to discern possible LQG imprints in the characteristics of regions close to BHs horizons and particularly within the idea to constrain the underlining graph features. Constraints are provided as restrictions of the graph features with respect to ADM and polymeric mass (M,m), the (ϵ,P) polymeric metric parameters, and the minimal LQG area parameter $a_{o}$. (Eventually, we enlarged the parameters’ value ranges to test the model, bracing the hypothesis of interacting attractor in an astrophysical BH scenario, where the attractor, and consequently the graph, eventually may evolve—inducing a transition from a point to another on a bundle in the extended plane). Discrepancies are highlighted by the comparative analysis with the reference solution of the general relativistic onset. As metric bundles are particular sets of BH solutions which are defined by properties of special associated light surfaces, this issue has been addressed with the investigations of the properties of the orbital null like frequencies ω (characteristic bundle frequencies—all the geometries of the bundle *per*-definition have equal values of orbital light frequency). Particularly, we are interested in the properties of light-like limiting frequencies of stationary observers tracing some characteristics of the regions close to the horizons in the sense of the extended plane and the replicas. A goal of this analysis was to provide constraints to the graph construction, which, in various ways, underlines the space(-time) structure of the model bridging the classical limit and quantum theory. In this respect, this analysis actually was inspired by the idea to relate the graph to light propagation, within Killing horizons and thermodynamics through the MB formalism. Besides the problem of the observation of the possible quantum effects on large (macroscopical) scale structure, there are on the theoretical grounds several aspects related to these theories to be clarified. The graph model quite naturally encodes the geometry discretization with the loop quantum gravity states. The key point of these quantum gravity approaches is actually the geometrical interpretation—in other words, to assign an opportune and adapted geometry to (the set of LQG) states. LQG states (quanta of space) were provided as spin network states, which are associated with a graph for the 3D (quantum) geometry. (The graph granulates the geometry, constituents and structure; therefore, there is large interest in the analysis of this very fundamental idea beyond a novel gravity geometrization/universalism). A second key question is of course to reconcile the quantum approach to a classical or (semi-classical) continuous geometry. The different regimes of the theory are provided generally by the graphs. LQG is generally based on a fixed (lattice) graph replacing and “fine-graining” the texture of GR geometry and establishing part of the relational structure. The Hilbert space, formed by the states on the graph, provides a benchmarking between these. It should be also noted that indeed these special light-surfaces related aspects of GR causal structure (delimiting existence of static and stationary observers) to the graph geometry, in different parts of the same spacetime (through replicas) and different geometries (bundles). We also provided constraints considering possible thermodynamic transformations intended as a shift from one solution to another (a transition of horizontal lines of the extended plane). We focused in this investigation particularly on the analysis of the BH thermodynamical properties considering luminosity and surface gravity. In the MB frame, we found variations of these quantities on the bundle curves, and thus relate the different geometries, according to the metric parameters, with their thermodynamic characteristics.

***2.*****MBs for LQG-BH solutions.** This analysis ultimately also clarifies aspects of MBs introduced in [6,7,8,9,10,11] when applied to the spherically symmetric case generalizing the tangency conditions of MBs with the horizons’ curves in the extended plane with a notion of approximations in the sense of Figure 5. As a sideline of this analysis, the MBs approach provided a novel frame of analysis and representation of the families of geometries with Killing horizons. This reinterpretation started from the construction of the extended plane for LQG polymeric BHs solutions in Section 2.1.2 comparing with the case of the Reissner–Norström geometry in Section 2.2, whereas, in Section 3, we introduced the metric bundles of the LBHs. One goal was to explore the MBs for the spherically symmetric static solutions as limiting solutions in the extended plane, hence the comparative analysis with the extended plane in Kerr geometries and the analysis with RN geometries. For one side, the metric (1) has similarities in RN spacetimes. On the other side, we used the electrically charged and spherically symmetric spacetime to enlighten properties of the extended plane. In the extended plane, the RN geometries were interpreted in [6] as limiting geometries of the (stationary electro-vacuum) Kerr-Newman solution occurring when the total charge is $Q_{T}=Q/M$. In this respect, the RN geometry was seen as a point in the Kerr-Newman extended plane of P−r/M, where P is pair of two parameters.

***3.*****MBs and BHs thermodynamics.** The analysis has been completed with the re-formulation in Section 4 of several aspects of the BH thermodynamics, where particularly in Section 4.1 the BHs surfaces gravity $\kappa_{\pm }$, the luminosity *L*, and the temperature $T_{\mathrm{BH}}^{\pm }$ have been investigated in terms of the loop model parameters P, thus these quantities are considered on metric bundles. Relevant for emission analysis is the region r∈[2M,3M], which is in the limiting Schwarzschild geometry (*M* is the ADM mass) the region between the outer horizon and the last photon circular orbits, which here has a special role in the bundles analysis as we showed in Figure 6. In conclusion, we constrain the graph-metric bundles and thermodynamics. The notion of horizons’ replicas provided by the collections of the MBs allows for reinterpreting the (classical) thermodynamical BH physics in terms of transitions from one point to another (on the vertical line) of the extended plane. From this standpoint, we focused on the explorations of the main quantities of LBHs physics entering into the analysis of BH thermodynamical transformations.

***4.*****Astrophysical relevance and phenomenological impact.** A further goal of our work was therefore also to test the MBs in the context of modified gravity. The goal is ultimately to detect (and interpret) the hints of quantum modifications outside the horizons, constraining eventually the graph properties—here the parameters P we used to construct the extended plane or the masses (the loop and ADM mass), and to evaluate a possible shift in the model parameters evolving from one solution in the extended plane to another. Our analysis presents an observational frame provided by the MBs onset grounded on the analysis of certain light-surfaces of the geometries. These light surfaces are the basis of several constraints of aspects of accretion disks. The characteristic bundles frequencies and connects regions close to the horizons to far regions. A further advantage of this method with respect to others (for example, the analysis of particle motions or spectra emission) is for its astrophysical interests, opening a wide window of different applications centered on the concept of MBs. The definition of these particular surfaces and their associated orbital frequencies are the basis of constraints to different results of the High Energy Astrophysics of BHs, providing therefore a powerful and ample investigation scenario. Up to now, no observational evidence has outlined a clear distinctive signature of quantum gravity scenario but on constraints on the existing proposals. There is therefore a great deal of attention on noticing any discrepancies in the current observations with respect to the predictions of the standard theoretical setup enclosed in GR theory that could be possibly explained in a quantum model. In this sense, the astrophysical setting offers the most natural arena for investigating the phenomenology of quantum gravity; in particular, one can search for new phenomena, which are unpredicted by the current GR model, but explained in a quantum gravity framework. Such observation could provide a strong constraint on the validity of many models. In this analysis, by comparing the predictions of the model with the features of the ordinary general relativistic astrophysics, within the analysis of these light-surfaces and the derived concept of metric bundles, we highlight some small but finite discrepancies, expectably detectible from the observations. In this perspective, the construction of the extended plane and metric bundles, with the replica definition, has consistently proved the possibility to detect the existence of divergences from expected prediction of the GR model of reference. Several observational channels are opened today in this context applicable to the examination of the light-surfaces; for example, we mention the recent window of Gravitational Wave analysis and especially the Event Horizon Telescope to explore from different (independent) angles the physics of BHs and their horizons. We tested the viability of this method to constrain the theory and possible observational evidence on the Astrophysical phenomena related to the BH physics focusing on the BH events’ horizon analysis. For these reasons, this investigation obviously could not exclude considerations on BH thermodynamics, directly governed by the BH horizon. The discrepancies highlighted between the predictions of these models, and the general relativistic ones are small but may be detectable.

In conclusion, the light-surfaces are at the base of MB definitions, as their characteristic frequencies have a wide field of application in many different aspects of BH astrophysics considering magnetic fields and different features of accretion physics. Therefore, we believe this approach could be a fruitful environment in which we can highlight the details attributable to transition from classical to quantum scales, characterized by non-trivial modification from the corresponding GR counterpart. It can be expected that this departure from the results can be evident also in the extended matter configurations, as accretion disks, their dynamics, and morphology. We expect therefore to apply this method in different exact and approximated solutions.

## Figures and Tables

**Table 1:** Lookup table with the main symbols and relevant notations used throughout the article with a brief description and reference to the first introduction of the term. Links to associated sections, definitions, and figures are also listed. Notation RN refers to Reissner–Norström geometry. We specify that $\sigma \equiv \sin^{2}\theta$ and $H\left(r\right)=g_{\phi \phi }/\sigma$. In general, we adopt notation $Q_{•}\equiv Q\left(r_{•}\right)$ for any quantity Q evaluated on a general radius $r_{•}$. A notable example concerns the case of quantities $Q_{\pm }\equiv Q\left(r_{\pm }\right)$ evaluated on horizons $r_{\pm }$ where we use superscript (occasionally subscript where necessary) ± respectively and, for convenience with the common use in literature and specified in the text, we use $Q_{H}\equiv Q_{+}$ for quantities evaluated on the outer horizon $r_{+}$. The frequency notation is excluded from this rule: $\omega_{\pm }$ are limiting photon orbital frequencies which on the horizons of the spherically symmetric geometries considered here are clearly null or $\omega_{\pm }\left(r_{\pm }=0\right)$.

$\left(\xi_{t},\xi_{\phi }\right)$	Killing fields of the geometry	Equation (12)–Section 2.1 and Section 3
$a_{o}=A_{min}/8\pi$,	the area parameter	Equation (2)–Section 2.1
$A_{min}$	minimum area gap of LQG	Equation (2)–Section 2.1
*P*	metric polymeric parameter	Equation (2)–Figures 1 and 2–Section 2.1
$\epsilon =\gamma_{BI}\delta$	δ = metric polymeric parameter, $\gamma_{BI}$ = Barbero–Immirzi parameter	Equation (2)–Figures 1 and 2–Section 2.1
*M*	ADM mass in the Schwarzschild limit	Equation (2)–Section 2.1
*m*	mass polymeric parameter function	Equation (2)–Figures 1 and 2–Section 2.1
$r_{\pm }$	horizons	Equation (3)–Figures 1 and 2–Section 2.1
$\epsilon_{\pm }$	horizons in ϵ-loop parameter	Equation (6)–Figure 2
$P_{\pm }$	horizons in *P*-loop parameter in extended plane	Equation (4)
$L=\partial_{t}+\omega \partial_{\phi }$	null Killing vector (generators of Killing event horizons)	Section 2.1.1
$L_{N}=0$	Killing vector L norm g(L,L)	Equation (12)–Section 2.1.1 and Section 3
$\omega_{\pm }$	light-like ($L_{N}=0$) limiting frequencies for stationary observers	Equation (9)
$\omega_{Sch}$	limiting frequencies for the Schwarzschild geometry	Equations (9) and (23)
$Q_{T}$	RN spacetime “total charge”	Equation (8)–Section 2.2
$a_{\pm }$	Kerr Killing horizon curve in the extended plane	Section 2.2
$Q_{\pm }$	RN horizon in the extended plane	Section 2.2–Figure 2
$Q_{\omega }$	RN metric bundles	Equation (9)
$r_{a_{o}}^{min}=\sqrt{a_{o}}$	a minimum curve for the $\sqrt{H(r)}$ as function of *r* ($\sqrt{H(r_{a_{o}}^{min})}=2r_{a_{o}}^{min}$)	Figure 4–Section 2.1
$\sigma_{\omega }$	metric bundles:θ parametrization	Equation (27)
$a_{o}^{\pm }\left(m,P\right)$	metric bundles: $a_{o}$-parametrization	Equation (28)–Figure 11
$r_{\tau }$	solution of $\partial_{P}\omega_{\pm }=0$	Equation (26)–Figure 9
$m_{\tau }$	solution of $\partial_{m}\omega_{\pm }=0$	Equation (24)
$\kappa :\nabla^{\alpha }L=-2\kappa L^{\alpha }$	(acceleration) on $r_{\pm }$, $\kappa_{\pm }$ define BH surface gravity	Equation (36)–Section 2.1.1 and Section 4.1
$T_{\mathrm{BH}}$	BH temperature	Section 2.1.1 and Section 4.1
$A_{\mathrm{BH}}$	BH areas	Equation (31)–Figure 12–Section 2.1 and Section 4.1
L(m)	Luminosity	Equation (42)–Figure 15–Section 4.1

**Table 2:** Quantities $a_{o}^{i}$ for i∈{1,…,7} represented in Figure 14. $a_{o}$ is the minimal loop areas, functions $a_{o}^{i}$ are extremes of the surfaces areas $\kappa_{\pm }$. *M* is the ADM mass, *P* is the polymeric function, and *m* is the polymeric mass.

$a_{o}^{1}\equiv \frac{4M^{2}P^{4}}{\sqrt{3}{\left(P+1\right)}^{4}},$	$a_{o}^{2}\equiv \frac{4M^{2}}{\sqrt{3}{\left(P+1\right)}^{4}}$
$a_{o}^{3}\equiv \frac{4M^{2}P^{4}}{{\left(P+1\right)}^{4}}\sqrt{\frac{\left(P-2\right)}{\left(3P-2\right)}},$	$a_{o}^{4}\equiv \frac{4M^{2}}{{\left(P+1\right)}^{4}}\sqrt{\frac{\left(2P-1\right)}{\left(2P-3\right)}},$
$a_{o}^{5}\equiv 4m^{2}P^{4}\sqrt{\frac{\left(P^{2}-2\right)}{3P^{2}-2}},$	$a_{o}^{6}\equiv \frac{4m^{2}P^{4}}{\sqrt{3}},$
$a_{o}^{7}\equiv \frac{4m^{2}}{\sqrt{3}}$	
